# Transparent Photothermal Slippery Surface Based on Monolayer Self‐Assembled MXene Film for Anti‐Fogging and De‐Icing

**DOI:** 10.1002/advs.202522420

**Published:** 2026-02-27

**Authors:** Xiao Han, Yiming Xie, Mingjia Sun, Di Zhao, Kesong Liu, Liping Heng, Lei Jiang

**Affiliations:** ^1^ State Key Laboratory of Bioinspired Interfacial Materials Science Bioinspired Science Innovation Center Hangzhou International Innovation Institute Beihang University Hangzhou China; ^2^ State Key Laboratory of Bioinspired Interfacial Materials Science School of Chemistry Beihang University Beijing China

**Keywords:** anti/de‐icing, photothermal conversion, self‐assembly, slippery surfaces, transparency

## Abstract

Transparent surfaces with autonomous anti‐fogging and de‐icing capabilities are critical for smart windows, eyewear, and optical sensors. Existing solutions relying on wettability engineering or bulk photothermal materials suffer from poor transparency, contamination vulnerability, or energy inefficiency. Here, we report an ultrathin (2.5 nm) MXene film self‐assembled at liquid‐liquid interfaces via Marangoni flow, achieving 82.5 % visible transparency while enabling a high photothermal conversion effect (ΔT∼25.1°C ± 2.9°C) under 100 mW cm^−2^. It is due to the unique percolative MXene network formed at ultralow loading (< 0.1 mg cm^−2^), leveraging the critical percolation thresholds to reconcile ultraviolet (UV, 300–400 nm) and near‐infrared (NIR, 700–2000 nm) absorption with visible transparency (82.5 %, 400–700 nm), which overcomes the trade‐off between solar harvesting and transparency. Therefore, coupled with a silicone oil‐infused slippery surface, the composite coating (TPSS) exhibits rapid ice shedding (85 s at −20°C under 1 sun) and fog resistance (at 90 % humidity). Outdoor demonstrations (Beijing, 2.1°C) on eyewear and architectural models validate frost suppression, rapid de‐icing, and mechanical flexibility. This scalable, sunlight‐powered platform bridges transparency and icephobicity for next‐generation optical devices.

## Graphical abstract

The ultrathin (2.5 nm) MXene film self‐assembled at liquid interfaces breaks the trade‐off between transparency (82.5 %) and photothermal conversion effect (DT∼25.1°C ± 2.9 °C) under 100 mW cm^−2^. After encapsulated by a slippery surface with low adhesion, this transparent film can be applied in smart windows, eyewear, and optical sensors to autonomous anti‐fogging and de‐icing under sunlight.

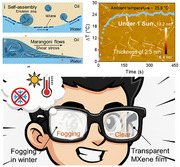

## 1 Introduction

Envisioning future smart technologies, multifunctional glass panels capable of intelligent display [1], autonomous thermal management [2, 3], and self‐cleaning [4] are poised to become indispensable in daily life and transportation [5]. Among these, transparent panels with autonomous de‐icing and defogging capabilities are particularly vital in high‐latitude winter conditions, providing reliable solutions for building insulation, safe driving, and anti‐fogging protection for eyewear and optical sensors [6]. Market analyses forecast that by 2027, the global market for highly transparent anti‐icing and anti‐fogging materials will surpass 10 billion RMB ($1.5 billion USD) [7]. Although conventional de‐icing methods–such as mechanical scraping, electrical heating, and microwave irradiation–can mitigate ice and avoid snow accumulation, these approaches are often time‐consuming, energy‐intensive, and lack real‐time adjustability, hindering their practical deployment [8, 9, 10]. To address these limitations, recent efforts have focused on combining low‐adhesion surfaces (such as superhydrophobic or slippery surfaces) with photothermal materials to achieve passive solar‐driven de‐icing and self‐cleaning without energy consumption [11, 12, 13, 14]. However, the fundamental requirement for smart panels'–high visible transparency–often conflicts with efficient photothermal anti‐icing. Superhydrophobic or slippery coatings, typically fabricated via micro/nano‐structuring, tend to scatter and reflect visible light, thereby reducing transparency [15, 16]. Meanwhile, effective photothermal conversion requires strong light absorption to generate heat, yet high transparency demands minimal absorption in the visible spectrum(400–700 nm), resulting in an intrinsic trade‐off between optical transparency and photothermal performance [17, 18].

To address this dilemma, a promising strategy involves engineering the material's absorption spectrum to maximize absorption in the near‐infrared (NIR, 700–2000 nm) and ultraviolet (UV, 200–400 nm) regions while keeping visible light transportation [19, 20]. Solar radiation reaching Earth's surface contains only 45.5 % visible light, with NIR and UV regions contributing 49.9 % and 4.6 %, respectively, making this approach thermodynamically favorable [21]. However, conventional strategies–primarily embedding nano‐photothermal materials such as carbon‐based fillers [22], metal nanoparticles [23], or semiconductors [24] into transparent polymer matrices–often fail to achieve broadband absorption across the entire 200–2000 nm range. This limitation arises because isolated nanofillers in polymer substrates rely on localized surface plasmon resonance (LSPR) [25, 26], molecular thermal vibrations [27, 28], or non‐radiative coupling mechanisms [29, 30] that are inherently confined to narrow wavelength bands [31]. Broadband absorption including UV–vis–NIR regions (200–2000 nm) emerges when the nanofiller concentration exceeds the percolation threshold to replace the plasmon coupling at a single resonance frequency [21]. However, the high nanofiller loading required to reach this threshold with obvious random dispersion severely compromises transparency. To reduce the percolation threshold, surface‐coating techniques, including spray‐coating [32], spin‐coating [33], sputtering [34], and chemical vapor deposition (CVD) [35], have been employed to concentrate photothermal nanomaterials within thin surface layers. Nevertheless, these methods frequently produce non‐uniform films with uncontrolled thickness (> 100 nm) or nanoparticle aggregation, ultimately degrading either transparency or photothermal performance. Only a few studies have achieved high energy absorption with visible translucence, and these typically rely on narrow‐band absorption or require micron‐thick coatings, failing to fundamentally resolve the trade‐off between transparency and photothermal conversion [36].

Here, we present a liquid interfacial self‐assembly technique for creating monolayer‐bridged MXene films with a thickness as low as 2.5 nm through Marangoni‐flow‐driven assembly [37, 38, 39, 40, 41]. The inter‐bridged MXene network achieves efficient electrical percolation at a low threshold, facilitating rapid photothermal conversion (ΔT∼25.1°C ± 2.9°C) under solar illumination (100 mW cm^−2^). Simultaneously, the spectral analysis reveals that these films display strong broadband absorption in both UV and NIR regions (200–400 nm and 700–2000 nm), while maintaining high visible transparency (82.5 %, 400–700 nm). It is believed that the contribution of NIR and UV regions to temperature rising accounts for 62 %, effectively decoupling energy conversion from visible light transmission. By integrating this ultrathin MXene layer with a transparent slippery surface based on oil‐infused PDMS oleogel [42], we create a robust, optically transparent, and low‐adhesion surface. This transparent photothermal slippery surface (TPSS) not only harnesses solar energy for autonomous antifogging and self‐heating but also facilitates rapid self‐cleaning and passive de‐icing under harsh outdoor conditions (e.g., −20°C). The flexibility brought by the ultra‐thin thickness makes it possible to attach to various shapes of substrates. This integrated photothermal slippery platform holds promise for a wide spectrum of intelligent optical and architectural applications, ranging from smart windows and automotive glass to prescription lenses, sports eyewear, and environmental sensors.

## 2 Results and Discussion

### 2.1 Design of the Monolayer Self‐Assembled MXene Film

**FIGURE 1:**
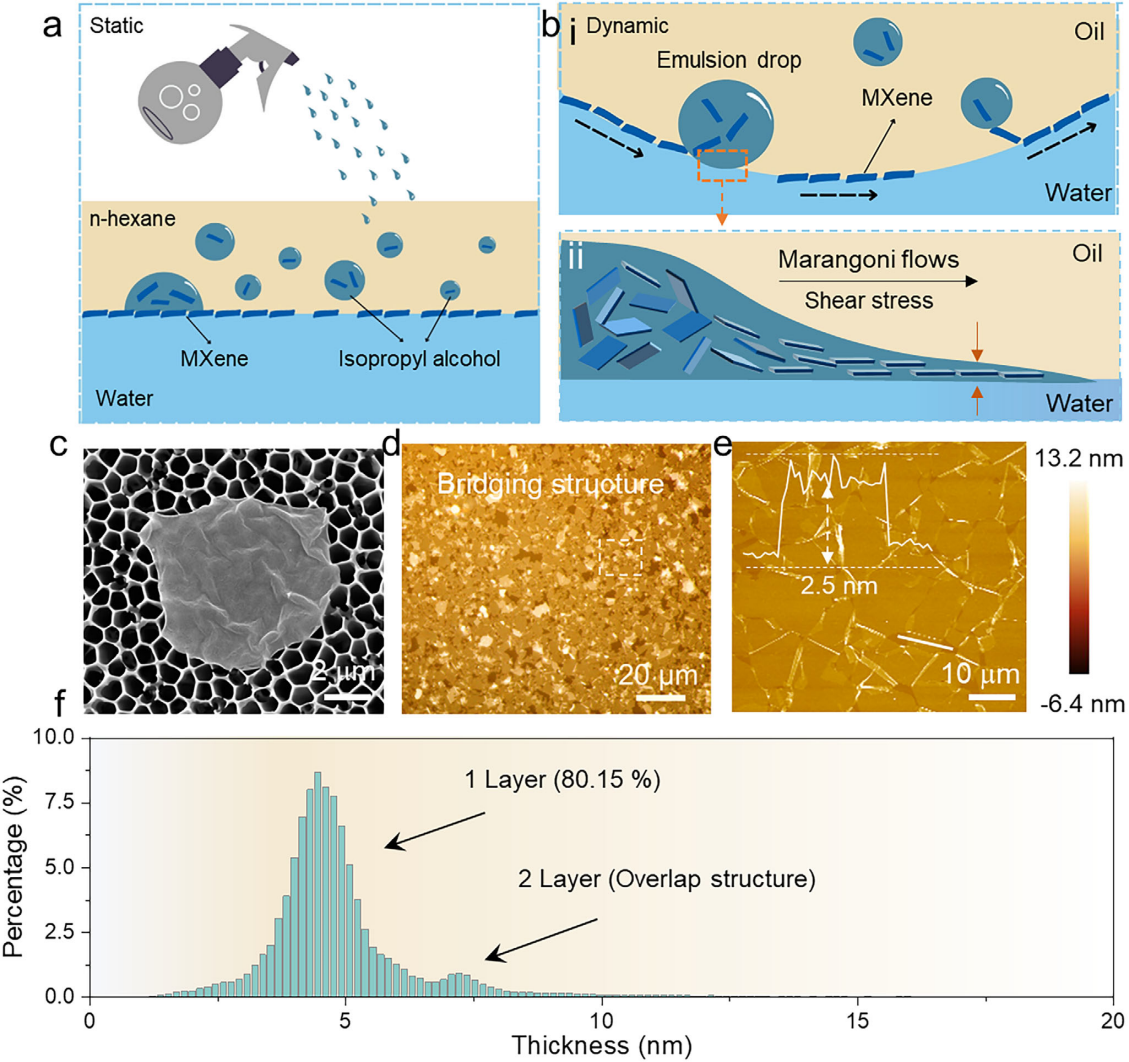
Design of the self‐assembled Mxene film with bridging structure. (a) Schematic illustration of the liquid–liquid interfacial self‐assembly of 2D MXene nanosheets. (b) Dynamic illustration of the self‐assembly process of MXene nanosheets driven by the Marangoni effect. Subpanel (i) shows the dynamic behavior of the MXene suspension prior to contacting the water phase; subpanel (ii) depicts the Marangoni flow and nanosheet self‐assembly upon the instant the isopropanol‐based MXene dispersion meets the water phase. (c) Scanning electron microscopy (SEM) image of exfoliated MXene nanosheet. (d) Optical microscopy image of a monolayer MXene self‐assembled film, where the light‐colored MXene sheets are uniformly oriented on the brown substrate. (e) Atomic force microscopy (AFM) image of the monolayer MXene self‐assembled film. The inset image is the height profile of the monolayer MXene (2.5 nm) in a self‐assembled film. (f) Height profile statistics of the monolayer MXene self‐assembled film in AFM image (e).

To maximize photothermal conversion while preserving optical transparency, adjusting the material's absorption bandwidths to achieve broadband absorption in the NIR/UV while suppressing visible absorption is necessary. Previous studies on Au–TiO_2_ coatings have demonstrated that electrical connectivity within the film enables strong broadband absorption and converts it into thermal energy. Therefore, to enhance electrical percolation at exceptionally low filler concentrations, a prerequisite for maintaining high optical transparency, we employed a liquid interfacial self‐assembly approach. As shown in Figure 1a,b, self‐assembly of the 2D nanosheets is achieved under the action of Marangoni by taking advantage of the surface tension gradient between different solvents (water/isopropanol).

Specifically, Ti_3_C_2_T_x_, as the prototypical MXene material in the 2D family (Figure S1), exhibits exceptional broadband optical absorption owing to its exceptionally high free carrier density (∼10^22^ cm^−3^), rich functional groups on the surface, and the surface plasmon resonance effects (Figure S2). Therefore, to achieve monolayer assembly of 2D MXene, the exfoliated single‐layer Ti_3_C_2_T_x_ (with an average lateral size of 3.17 µm, Figure 1c; Figure S3) is first dispersed in isopropanol. Upon spraying the dispersion onto the hexane‐water interface, the surface tension gradient between isopropanol and water induces Marangoni flow, at which the associated tangential stress promotes the rapid spreading of spherical droplets along the oil–water interface (Figure S4). During this spreading process, the normal stress at the oil–water interface further facilitates the orientation and monolayer assembly of 2D nanosheets in isopropanol until encountering neighboring flakes or container boundaries. Remarkably, just tens of droplets (2 mg mL^−1^ MXene in isopropanol) are sufficient to cover an area of 10 cm^2^ (Figure 1d), significantly raising the percolation threshold of the MXene film while greatly reducing both material consumption and fabrication cost (Movie S1). It is worth noting that as the concentration of MXene solution increases (from 1 to 3 mg mL^−1^), the coverage of MXene nanosheets gradually becomes more compact. Yet when the concentration is above 3 mg mL^−1^, we observed pronounced local stacking/aggregation of MXene nanosheets, which reduces the optical transmittance and may introduce scattering. Therefore, with the suitable MXene concentration (2 mg mL^−1^), the self‐assembled monolayer MXene film is only 2–4 nm thick, ensuring high optical transmittance in the visible range (Figure S6). As shown in Figure 1e, the MXene nanosheets in the self‐assembled film exhibited an ultrathin thickness of 2.5 nm, proving the single‐layer self‐assembly. Detailed statistical analysis of the self‐assembled structure reveals that approximately 80.15 % of MXene nanosheets are arranged in well‐oriented monolayers, while a minority of stacked multilayers serve as effective bridges (4.2 nm, Figure 1f; Figure S6), thus enhancing the electrical connectivity of the MXene nanofilm. It is believed that the ultrathin films with only partially stacked structure not only ensure the haze and transparency of TPSS (haze < 4 %, Figure S7), but also guarantee the electrical percolation (Figure S8). Consequently, this interconnected MXene framework greatly enhances interband transitions, surface plasmon resonance, and broadband light absorption, leading to superior photothermal conversion efficiency.

### 2.2 Optical Properties of Self‐Assembled MXene Films

**FIGURE 2:**
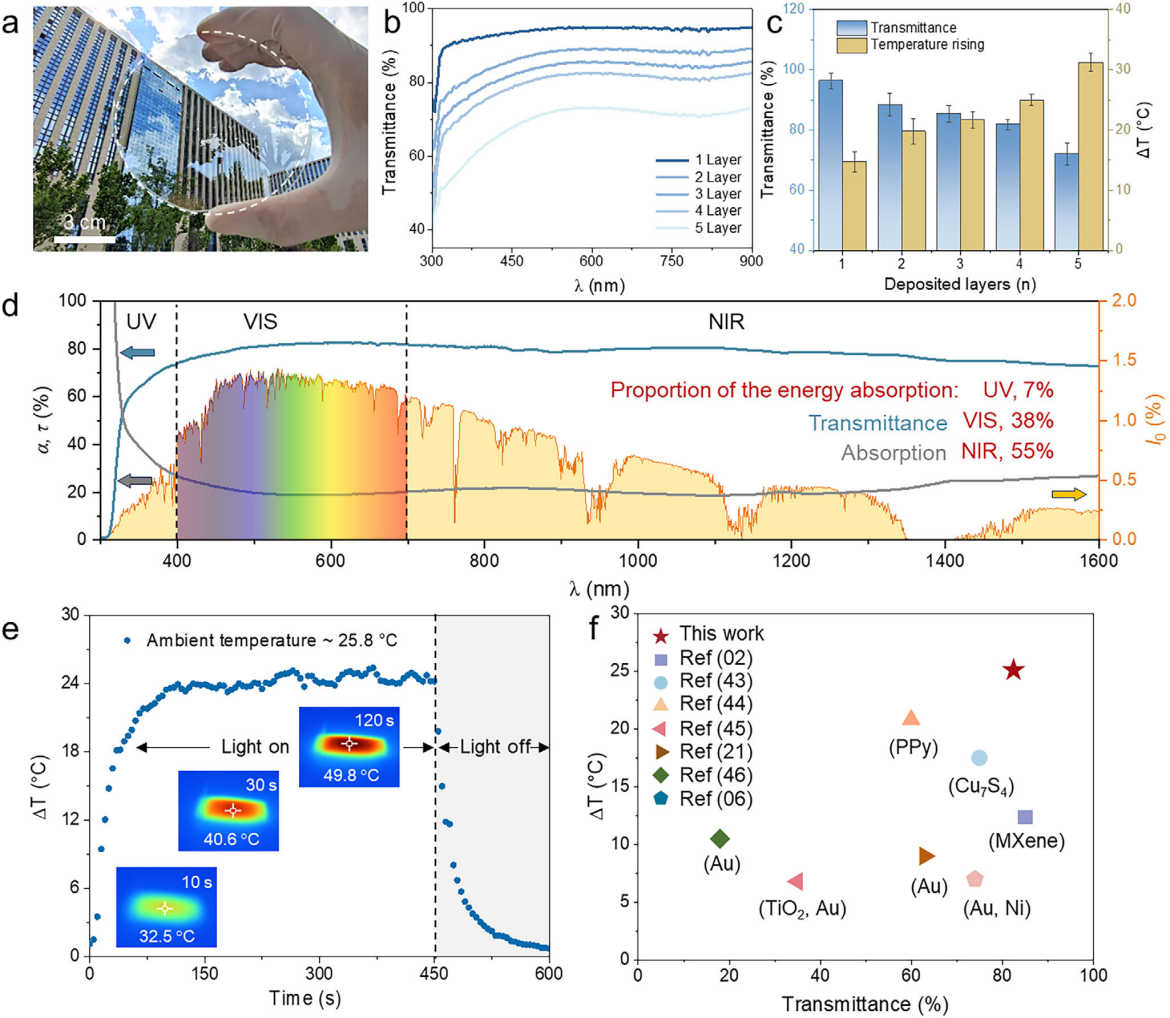
Optical properties of self‐assembled MXene films. (a) Optical image of the ultrathin MXene photothermal film coated on a glass substrate, demonstrating its exceptional transparency. (b) Transmittance spectra in the visible range for MXene films with varying numbers of stacked layers. (c) Variation of optical transmittance and photothermal conversion performance as a function of the number of MXene film layers. Error bars in (c) are calculated from the five measurements. (d) Optical transmittance (τ) and absorbance spectra (α) of MXene films across the full range (UV–vis–NIR). The yellow area indicates the energy distribution of the standard solar spectrum. The integral of the absorption curve shows that the energy absorption of TPSS in different bands (UV–vis–NIR) accounts for 7 %, 38 %, 55 %, respectively, effectively utilizing the absorption of UV and NIR light while preserving optical transparency (VIS band). (e) Photothermal temperature rise curve of a highly transparent MXene film (three layers) under one‐sun illumination. Upon removal of the light source, the film temperature decreases accordingly. (f) Comparison of the optical transmittance and photothermal conversion efficiency of self‐assembled MXene films (two layers) with the reported transparent photothermal coatings [2, 6, 21, 43, 44, 45, 46].

Owing to a single‐layer self‐assembled MXene layer (2–4 nm thickness), the composite assembled with transparent glass retains exceptional optical clarity. As shown in Figure 2a, scenery viewed through the MXene‐coated film remains remarkably clear and vivid, testifying to its superior transparency. Besides, UV–vis spectrophotometric analysis further reveals that the ultrathin MXene films deliver outstanding transmittance throughout the visible region. In particular, at a wavelength of 550 nm, the transmittance of the MXene film with a single layer can reach as high as 94.3 % (Figure 2b). Although sequential stacking of MXene films leads to a gradual decrease in transparency, three layers of MXene still achieve a transmittance exceeding 82.5 %. Even with up to five layers, the composite maintains more than 70 % transmittance (Figure 2c). Notably, while increased layer and film thickness inevitably reduce transparency, they simultaneously enhance photothermal conversion performance due to the better electrical connectivity (Figure S9). For instance, even a single layer of ultrathin MXene achieves a surface temperature increase of 14.8°C under one‐sun illumination (100 mW cm^−2^); when stacked into three layers (TPSS_3_), the temperature rise reaches 25.1°C ± 2.9°C, while still retaining 82.5 % transmittance—successfully balancing the competing requirements for high transparency and vigorous photothermal activity (Figure S10 and Movie S2).

To illustrate the mechanism of the high transparency and photothermal perpority, full‐spectrum analysis (UV–vis–NIR, 200–2200 nm) uncovers the basic rationale for this synergy: the MXene film exhibits ultrahigh VIS transmittance (> 80 %) alongside unexpected broadband absorption in UV (α = 89 %) and NIR (α = 26 %) regions (Figure 2d). When MXene's percolative network exceeds its critical percolation threshold, interflake tunneling transitions dominate over visible‐region interband absorption. This shifts localized plasmon resonance into delocalized broadband absorption above the percolation threshold, without pronounced narrow peaks, closely resembling dense percolated MXene films whose response has been modeled as a heavily damped Drude conductor with strong broadband photothermal conversion. In other words, dense, percolated MXene networks effectively behave as “lossy metallic films”: the strong electron scattering and interflake coupling provide broadband conductive losses, so that light over a wide spectral range can be efficiently converted into heat, instead of being transmitted or reflected. It is believed that the free electrons experience frequent collisions, leading to strong energy dissipation. As a result, the absorbed optical energy is rapidly converted into heat via electron–phonon scattering, rather than forming sharp plasmonic resonances at narrow frequencies. Thus, by integrating the energy absorption of the TPSS at different wavelength bands, we found that the energy absorption in the VIS region was only 38 %, while the energy absorption in the UV (7 %) and infrared region (55 %) was as high as 62 % (Figure 2d). As a result, under solar irradiation (1 sun, 100 mW cm^−2^), a transparent TPSS_3_ can achieve a rapid temperature rise of approximately 24.5°C within 120 s (Figure 2e), sufficient to quickly melt frost and ice layers on glass surfaces in winter, providing reliable anti/de‐icing capabilities. Compared to traditional fabrication approaches such as spray‐coating of noble metals or carbon‐based films, the liquid interfacial self‐assembly technique yields TPSS more than threefold improvement in photothermal efficiency over conventional photothermal materials (Figure S11). As shown in Figure 2f, compared with reported transparent photothermal coatings, the temperature rise and transmittance of TPSS show competitive performance. Note that even at low light intensity, the photothermal performance is still better than that of Au, Cu, and C films prepared by the spraying process (Figure S11). For example, the temperature rise of TPSS_3_ (with three layers of MXene film) can still reach 15.3°C ± 1.7°C, 20.1°C ± 2.6°C under 0.5, 0.8 sun, respectively (Figure S12).

### 2.3 Liquid Repellency Properties of the TPSS

**FIGURE 3:**
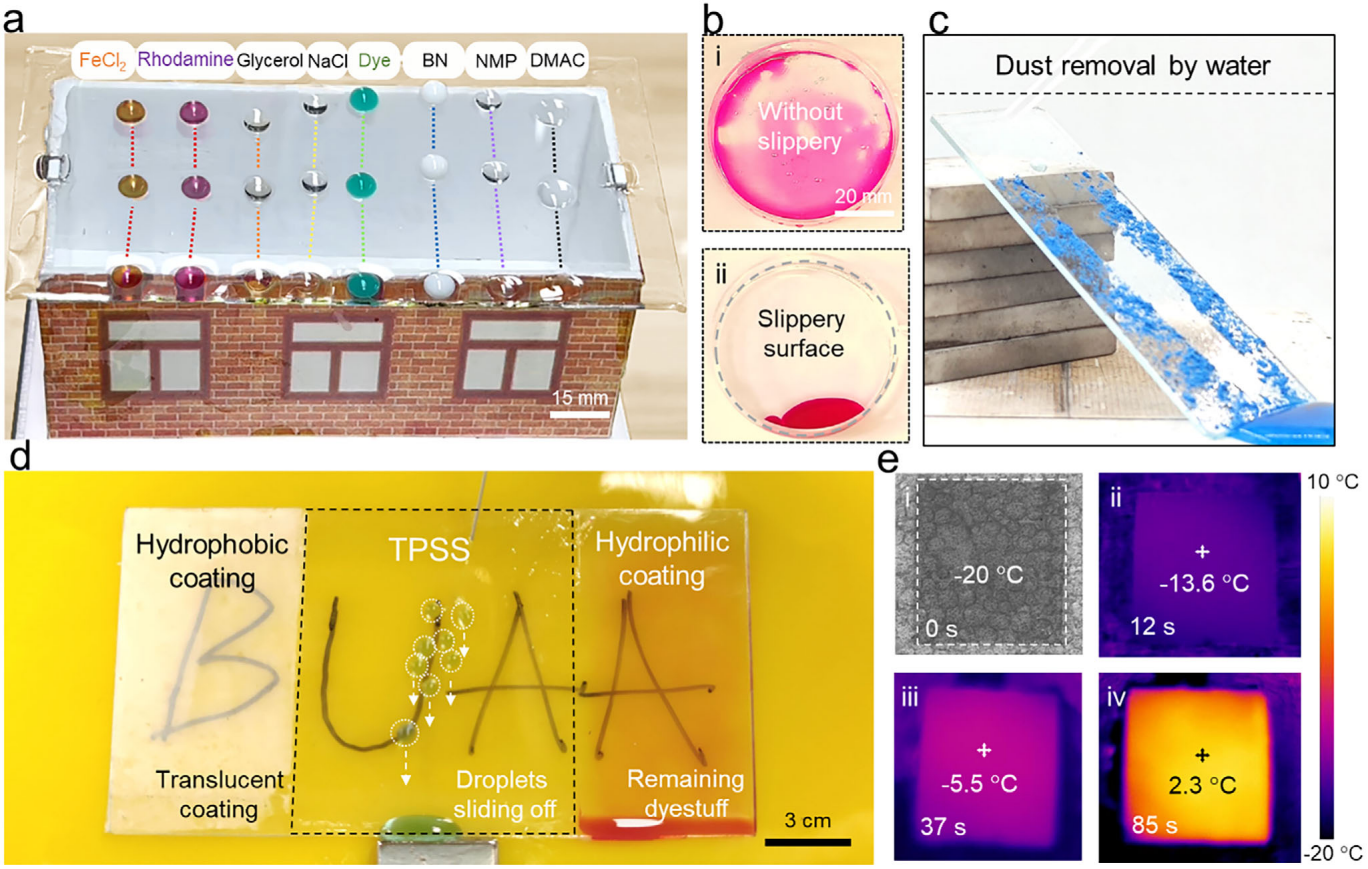
Liquid repellency properties of the TPSS. (a) Trajectories of various liquid droplets on the SLIPS (silicone oil‐infused PDMS), illustrating its outstanding low adhesion properties. (b) Optical images of Rhodamine solution droplets on a standard plastic dish and on a dish coated with PDMS oil gel, highlighting the antifouling performance of the SLIPS. (c) Optical images demonstrating the self‐cleaning performance of the SLIPS surface: dust particles are effectively removed as droplets slide down the interface. (d) Snapshots showing the translucent superhydrophobic coating, transparent TPSS with liquid repellence, and the polluted superhydrophilic coating on PMMA. (e) Optical images (i) and infrared thermal images (ii–iv) of PET films coated with TPSS_3_ (with three layers of the MXene film) under 1 sun at the ambient temperature of −20°C.

To achieve effective anti‐icing and anti‐fogging under low‐temperature conditions, it is essential not only to employ a photothermal conversion layer but also to incorporate a low‐adhesion surface that facilitates self‐cleaning of melted ice. Although conventional superhydrophilic surfaces have been widely used to prevent fog droplet formation, their inherently high surface energy makes them highly susceptible to contamination by organic pollutants, thereby severely limiting their long‐term effectiveness. To address this issue, we spin‐coated the surface of the MXene film with a highly transparent, lubricant‐infused PDMS gel to create the slippery surface. As shown in Figure 3a and Figure S13, this lubricant‐infused slippery surface enables rapid self‐cleaning of diverse liquid contaminants—including dye solutions, organic solvents, inorganic salt solutions, and nanoparticle suspensions—the droplets are swiftly removed by gravity within 20 s. By contrast, on conventional PET substrates without the SLIPS coating, dye molecules in aqueous solutions tend to remain on the surface, while the application of the transparent PDMS oil‐gel layer effectively prevents fouling (Figure 3b). In addition, even after exposure to substantial amounts of solid particulates, rapid self‐cleaning can be achieved by rinsing with water droplets, further maintaining the optical transparency of the material (Figure 3c). This exceptional performance is attributed to the low surface energy and self‐cleaning properties of the slippery surface. Unlike superhydrophobic surfaces that require micro‐ or nano‐structuring—often compromising optical clarity—the smooth SLIPS can be applied onto the photothermal layer without detriment to either transparency or photothermal conversion efficiency (Figure 3d). In addition, unlike the superhydrophilic surfaces with polluted dye, TPSS with the SLIPS shows excellent liquid repellency. This transparent, high‐performance TPSS thus offers dual functional advantages: anti‐fogging in low‐temperature outdoor environments and efficient de‐icing or defrosting at subzero temperatures. As demonstrated in Figure 3e, the TPSS_3_ can raise its surface temperature to 2.3°C within 85 s under a single sun illumination, even when exposed to an ambient temperature as low as −20°C. It is due to the ultralow average thickness of the PDMS coating (∼8 µm), although the thermal conductivity of bulk PDMS is limited to 0.15 W m^−^^1^ K^−^^1^. Therefore, photothermal heating generated by the MXene layer (23.8°C ± 0.4°C) is efficiently transferred to the outer PDMS surface (23.0°C ± 0.5°C) with negligible thermal drop (Figure S14).

In addition, the PDMS‑based slippery layer can act as a protective encapsulation to isolate from the erosion of oxygen and moisture, thus mitigating the moisture‐ and photo‑oxidation of internal MXene. It is believed that the photothermal temperature rise of TPSS samples remained without any noticeable degradation, and no delamination, corrosion, or loss of slipperiness was observed after being stored at relative humidities ranging from 10 % to 90 % (25°C) for up to 144 h (Figure S15). Moreover, continuous UV exposure at 365 nm with different lamp powers (5–25 W) for up to 720 min caused only minor fluctuations of ΔT (within ±1°C). Subsequently, we verified possible structural changes of MXene before and after environmental cycling to rigorously assess the stability of the TPSS. As shown in Figure S16a, the XRD of the bare MXene film after aging exhibits new diffraction peaks (25.3°, (101) planes of tetragonal anatase TiO_2_; 27.4°, (110) plane of rutile TiO_2_), indicating that the oxidized MXene contains predominantly anatase with a minor rutile fraction. In contrast, the XRD pattern of the MXene layer within TPSS after the same treatment shows no discernible anatase or rutile TiO_2_ peaks and remains essentially identical to that of the pristine MXene, confirming the stability of TPSS. This observation is fully consistent with the XPS characterization. Figure S16b presents the XPS Ti 2p spectra of bare MXene and PDMS‐encapsulated MXene after 144 h of environmental exposure. For bare MXene, the emergence of a dominant Ti4+peak (TiO_2_, 458.8 and 464.8 eV) and significant attenuation of the Ti─C bond (454.9 eV, intensity reduced by ∼62 %) confirm severe oxidation. In contrast, the TPSS exhibits negligible TiO_2_ peaks (atom <2.2 %), demonstrating protection of the slippery surface (oil‐infused PDMS): (1) physical barrier against H_2_O/O_2_ permeation and (2) chemical passivation via Si─O─Ti bonding at MXene edges. These results conclusively correlate structural stability with sustained photothermal performance. In addition, the systematic durability tests, including simulated rainwater impact and sand/dust abrasion, were carried out. As shown in Figure S17, we used a dripping setup (falling height ≈ 50 cm, flow rate ≈ 20 mL min^−^^1^) and defined one cycle as 10 min continuous impact followed by drying for the rainwater impact test. The temperature rise (ΔT) of TPSS remained almost unchanged at ≈ 25°C after 10 cycles, whereas a bare MXene film without PDMS protection quickly lost its photothermal response within 3–5 cycles due to structural damage and peeling. This poor durability of bare MXene mainly arises from its intrinsic hydrophilicity, chemical and mechanical vulnerability: (i) direct water impact and wetting can induce gradual oxidation/hydrolysis of Ti_3_C_2_T_X_, forming non‑conductive oxides and disrupting the conductive network; (ii) the ultrathin and rigid nanosheet network adheres weakly to the substrate and is susceptible to crack initiation and delamination under repeated hydrodynamic loading; and (iii) Hydrophilic MXene nanosheets will gradually dissolve and be carried away by the erosion of raindrops. A similar trend and mechanism were observed in the sand/dust impact test, where quartz sand (≈50–200 nm) was continuously dropped from 50 cm: TPSS maintained a stable ΔT (∼25°C) after 10 cycles, while the unprotected MXene film suffered severe abrasion, nanosheet removal, and almost complete performance failure. These results demonstrate that the slippery PDMS over‑layer not only physically shields the MXene from direct impact and abrasion but also isolates it from water and particles, thereby greatly suppressing oxidation, cracking, and delamination and imparting excellent mechanical durability to TPSS in simulated rain and sandstorm conditions.

### 2.4 Anti‐Icing and De‐Icing Performance of TPSS

**FIGURE 4:**
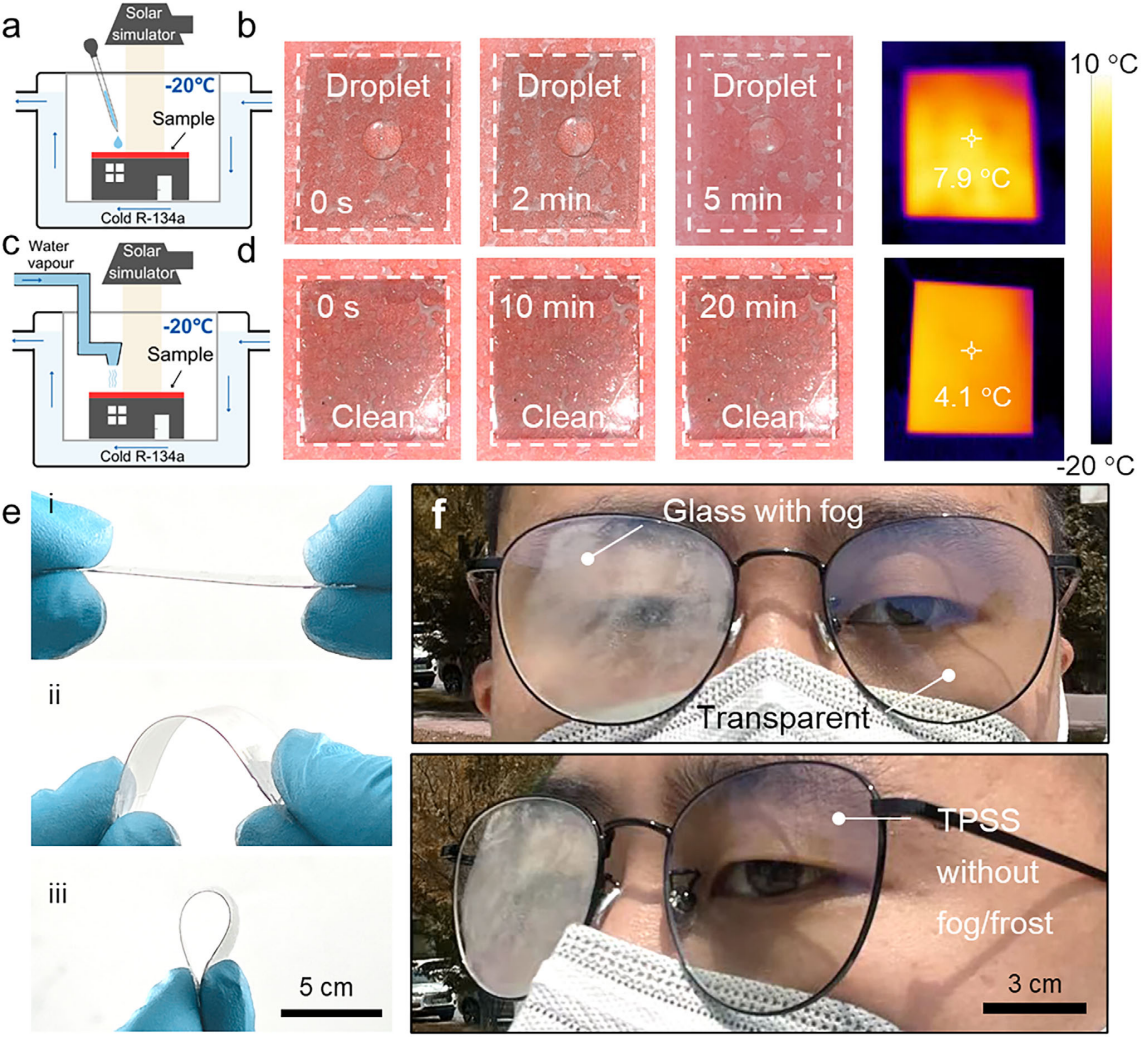
Anti‐icing and anti‐frosting/fogging performance. (a) Schematic illustration of the custom‐designed, low‐temperature environmental chamber equipped for controllable droplet deposition. (b) A sequential photographic series showing the behavior of supercooled water droplets upon deposition, together with the infrared thermal image of TPSS at −20°C under 1 sun illumination, demonstrates the exceptional anti‐icing performance. (c) Schematic diagram of the humidity‐controllable chamber with low‐temperature to verify the anti‐icing property. (d) Infrared thermal image of TPSS at −20°C under 1‐sun illumination, and sequential images illustrating its anti‐fog and anti‐frost capability under high‐humidity conditions (90 % RH). (e) Photographs of TPSS‐coated flexible PET films under various bending angles highlight their outstanding flexibility and mechanical durability. (f) Test images of TPSS‐coated antifogging eyeglasses in outdoor (I_0_ = 751 W m^−^^2^ in Beijing, 2.1°C). The left lens is coated with TPSS and maintains visibility even in the presence of upward humid exhalation from a mask, whereas the uncoated lens rapidly fogs up.

To evaluate the anti‐icing and anti‐fogging performance of TPSS_3_ under low‐temperature conditions, we constructed a custom environmental chamber. As illustrated in Figure S18, when liquid refrigerant R‐134a with ultralow temperature enters the evaporator directly connected to the chamber, it absorbs heat from the internal air and undergoes a phase transition, thereby reducing the chamber temperature to approximately −20°C. Once the temperature of the TPSS_3_ sample (with three layers of the MXene) equilibrates with the ambient environment, a xenon lamp is employed to provide vertical irradiation, simulating the intensity of 1 sun (Figure 4a). As shown in Figure 4b, under these conditions, the surface temperature of TPSS_3_ rapidly increases to 7.9°C within 5 min, even when the ambient temperature is around −20°C. At this stage, the deposition of a droplet of ice‐cold water (0°C) on the surface does not result in freezing. Furthermore, benefiting from the ultra‐low adhesion of the slippery interface, the unfrozen droplet can slide off swiftly after inclining the horizontal surface (Figure S19). In contrast, for samples coated with the oil‐gel alone (without the photothermal MXene layer), the dewetting nature of the surface can slightly delay ice formation, yet with continued exposure, residual droplets on the horizontal surface inevitably freeze (Figure S20). Moreover, TPSS_3_ demonstrates excellent antifogging properties in cold, humid environments by preventing the condensation of nanometer‐scale fog droplets or frost layers (Figure 4c,d). Owing to a remarkable temperature rise of 24.1°C under one‐sun irradiation, TPSS_3_ maintains a condensation‐free surface even in environments at −20°C and 90 % relative humidity (Figure 4d). By comparison, on slippery surfaces lacking the MXene layer, severe frost quickly accumulates and adheres persistently at low temperatures (Figure S21).

Notably, the ultrathin MXene films can be readily assembled onto flexible substrates as well as rigid ones. For example, when the self‐assembled MXene film is applied to flexible PET, the resultant membrane can be freely bent and deformed without damage (Figure 4e). As a result, TPSS_3_‐coated PET films can also be deployed on substrates that are difficult to coat by conventional methods, such as curved eyewear lenses or automobile windshields, enabling solar‐driven antifogging and self‐cleaning functionalities. For instance, applying a TPSS_3_ coating to eyeglass lenses provides effective, low‐power anti‐fogging even when exposed to warm exhaled air (∼37°C, >90 % humidity) during mask‐wearing outdoors (Beijing, 2.1°C, Figure 4f). In high‐latitude regions during the spring and winter, conventional lenses rapidly accumulate condensed microdroplets upon contact with exhaled breath, obscuring vision due to fogging. In sharp contrast, transparent lenses coated with TPSS_3_ not only prevent microdroplet condensation but also maintain optical clarity, thereby significantly reducing the risk of visibility‐related accidents.

**FIGURE 5:**
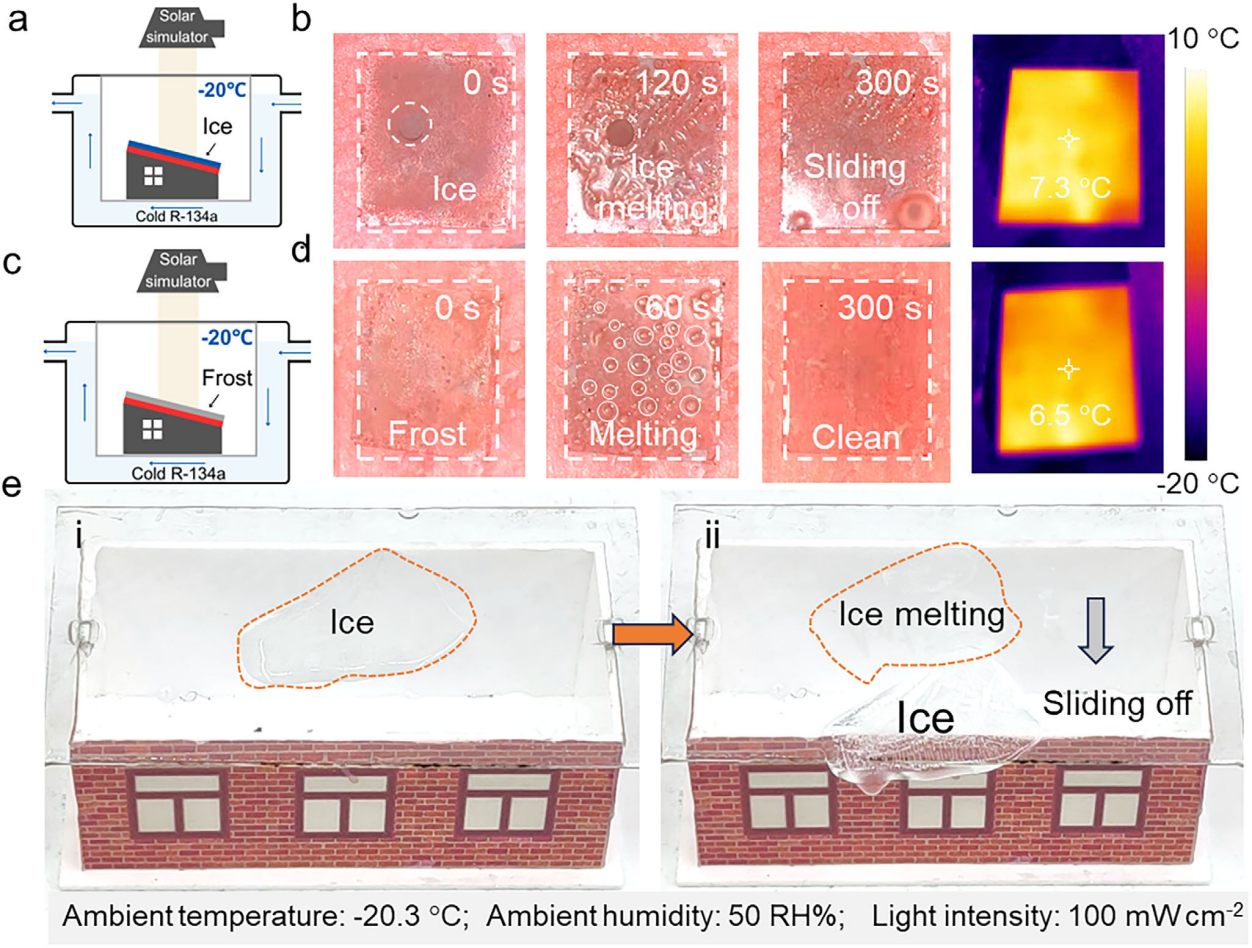
De‐icing and de‐frosting/fogging performance. (a) Schematic illustration of the custom‐built low‐temperature, constant‐temperature, and humidity environmental chamber. (b) Sequential optical images of ice crystal melting on pre‐frozen TPSS at −20°C under 1‐sun illumination, together with corresponding infrared thermal images of the interface temperature, demonstrate the material's excellent de‐icing performance. (c) Schematic illustration of the environmental chamber with low‐temperature to verify the de‐frosting property. (d) Sequential optical photographs of frost layer melting on pre‐frosted TPSS at −20°C under 1 sun illumination, along with corresponding infrared thermal images of the surface, highlight its outstanding defrosting capability. (e) Images of autonomous ice melting and de‐icing on a building model rooftop made from TPSS‐coated transparent PMMA, under low‐temperature conditions and 1‐sun illumination.

Beyond its anti‐icing and anti‐fogging capabilities, the ability of the photothermal ultra‐slippery surface to autonomously remove condensed ice or frost layers highlights the application of this novel anti‐icing material (Figure 5a). To demonstrate this feature, pre‐frozen TPSS samples were placed into the environmental chamber. Under 1 sun irradiation, the frozen ice layer atop the TPSS gradually melts as the surface temperature rises to 7.3°C, and within 10 min, the meltwater swiftly slides off the inclined, slippery surface—leaving behind a clean and clear substrate (Figure 5b). In contrast, PET substrates coated with only a slippery surface fail to melt surface ice even after 1 h of solar exposure (Figure S22). Similarly, when the TPSS is pre‐coated with a frost layer, it begins to melt within 5 min of illumination, leading to the formation of surface microdroplets (Figure 5c,d). These droplets are rapidly removed from the surface under gravity, facilitated by the ultra‐low adhesion of the slippery surface. This efficient self‐cleaning arises from the synergistic effects of the photothermal properties of the MXene film and the self‐cleaning performance of the slippery surface. Because of the slippery surface without a photothermal MXene film, the frost layer will firmly adhere to the material even on the inclined surface (Figure S23). To practically assess the effectiveness of the highly transparent TPSS in autonomous de‐icing applications for architectural glass, a transparent PMMA plate coated with TPSS was mounted atop a building model (Figure 5e). A 20 × 30 mm ice layer (approximately 0.5 cm thick), pre‐frozen onto the TPSS surface, was then exposed to simulated sunlight. Remarkably, the ice began to melt after about 5 min of illumination (Movie S3). Attributed to the low‐adhesion of the slippery surface, the entire ice layer rapidly slid off the surface before completely melting, leaving no liquid residue behind. These findings demonstrate the practical feasibility of utilizing solar‐driven, TPSS coatings for efficient, residue‐free de‐icing of transparent substrates in real‐world applications.

## 3 Conclusion

In summary, we demonstrate transparent anti/de‐icing materials that reconcile high photothermal conversion (25.1°C ± 2.9°C) with optical clarity (82.5 % transparency) via percolative MXene nanofilms. Marangoni‐driven interfacial self‐assembly achieves monolayer MXene networks at critical percolation thresholds, enabling broadband absorption (200–2000 nm). Attributed to the high absorption proportion (∼62 %) in UV (200–400 nm) and NIR (700–2000 nm) bands, the self‐assembled ultrathin MXene film with high transparence of 82.5 % in the VIS band exhibited a high temperature rise (∼25°C) under illumination of 100 mW cm^−2^. Therefore, coupled with a slippery surface, the composite TPSS has a rapid de‐icing property and anti‐fogging property (at −20°C), contamination resistance, and mechanical flexibility. It is believed that outdoor validations on eyewear and architectural glass highlight practicality in extreme climates. This work provides a scalable, energy‐autonomous solution for smart windows, automotive optics, and wearable devices, bridging the gap between transparency and icephobicity.

## Funding

This work was supported by the National Natural Science Foundation of China (22505054, 52273101, and 51922018), the Fundamental Research Funds for the Central Universities (KG21015201 and KG21020801), China Postdoctoral Science Foundation (2025M774222), and Postdoctoral Research Funding of Hangzhou International Innovation Institute of Beihang University (2025BKZ019).

## Conflicts of Interest

The authors declare no conflict of interest.

## Supporting information




**Supporting File**: advs74094‐sup‐0001‐SuppMat.docx.

**Supplemental Movie**: advs74094‐sup‐0002‐MovieS1.docx.

**Supplemental Movie**: advs74094‐sup‐0003‐MovieS2.docx.

**Supplemental Movie**: advs74094‐sup‐0004‐MovieS3.docx.

## Data Availability

The data that support the findings of this study are available from the corresponding author upon reasonable request.
