# Supplementary material for: Transparent Photothermal Slippery Surface Based on Monolayer Self‐Assembled MXene Film for Anti‐Fogging and De‐Icing
Source: Adv Sci (Weinh). 2026 Feb 27;13(20):e22420. doi: 10.1002/advs.202522420 (PMC13067774; doi:10.1002/advs.202522420)
Supplement: Supplementary file 1 — Supporting File: advs74094‐sup‐0001‐SuppMat.docx. [file ADVS-13-e22420-s001.docx]

Supporting Information

Transparent Photothermal Slippery Surface based on Monolayer Self-assembled MXene Film for Anti-fogging and De-icing

Xiao Han, Yiming Xie, Jiexin Hou, Mingjia Sun, Di Zhao, Kesong Liu*, Liping Heng* and Lei Jiang

State Key Laboratory of Bioinspired Interfacial Materials Science, Bioinspired Science Innovation Center, Hangzhou International Innovation Institute, Beihang University, Hangzhou 311115, China.

State Key Laboratory of Bioinspired Interfacial Materials Science, School of Chemistry, Beihang University, Beijing 100191, China.

Department of Mechanical Engineering, The Hong Kong Polytechnic University Hong Kong, Hongkong 999077, China.

E-mail: [henglp@buaa.edu.cn](mailto:henglp@buaa.edu.cn), liuks@buaa.edu.cn

1. Materials and Methods

**Materials**. Aniline, lithium fluoride (LiF) and isopropyl alcohol were purchased from Aladdin Reagent Co. Ltd. (Shanghai, China). Glycerol, glucose, methylene blue, sodium chloride (NaCl), absolute alcohol and epoxy resin were purchased from Sinopharm Chemical Reagent Co., Ltd. (Shanghai, China). Titanium aluminum carbide (Ti_3_AlC_2_) was purchased from Jilin 11 Technology Co., Ltd. Hydrochloric acid (HCl) was obtained from Yantai Fine Chemical Co., Ltd. N, N-Dimethylformamide and ethylene glycol was purchased from Guangfu Technology Development Co., Ltd. (Tianjin, China). Polydimethylsiloxane (PDMS) and dimethyl silicone oil (viscosity, 60 cSt) were purchased from Dow Corning (Midland, USA). Deionized water was produced by a deionized water system (DINEC, Hong Kong).

**Synthesis of the Ti_3_C_2_T_x_ flakes.** Ti_3_C_2_T_x_ flakes were prepared by etching process from MAX-phase, Ti_3_AlC_2_, following Gogosti’s work. Briefly, 1.6 g lithium fluoride was dissolved into 20 mL hydrochloric acid solution (9 M) in a centrifuge tube as the etchant solution. Subsequently, 1g Ti_3_AlC_2_ powders were etched with stirring at 40 °C for 36 h after being slowly added into the etchant solution. Etched MXene was washed twice with 1M hydrochloric acid solution, followed by being washed with deionized water. The washing process was repeated several times until the pH of the solution reached 6, and the sediment became sticky. The stripping process was executed by the turbine for 20 min to avoid oxidation. The solution was subsequently centrifuged for 30 min at 3500 rpm to collect the supernatant, which has a pronounced Tyndall effect.

**Fabrication of the TPSS.** The obtained supernatant with Ti_3_C_2_T_x_ flakes was mixed with anhydrous ethanol (volume ratio, 1:1) in a watering can. Pouring the hexane on top of DI water to set up an interface between these two liquids was the first step to form a uniform film. The Ti_3_C_2_T_x_ dispersion was then sprayed onto hexane. Due to the surface tension gradient, the Marangoni flow of these tiny droplets enabled the Ti_3_C_2_T_x_ flakes to spread along the interface until joint to others or the sidewall of the container, forming a continuous film. After removing the top hexane, the self-assembly film was transferred onto various substrates, such as PMMA, PET, glass, and annealed at 80 °C for 3 h. To improve the photothermal performance, multilayered MXene film was transferred onto the pyroelectric layer by repeating the above process. On the other hand, to reduce the adhesion force of the liquids to TPSS, a slippery surface was equipped on the top of the pyroelectric layer. Briefly, the PDMS precursor was first diluted by hexane to a concentration of 10 wt%, and then the dispersion was drip-coated on the lithium niobate wafer after mixing with the curing agent. The curing process was executed at room temperature for 5 h. Subsequently, a layer of dimethyl silicone oil was absorbed into the oil gel.

**Modulation of the optical and photothermal properties for TPSS.** To evaluate the optical property of the TPSS with different MXene layers, several TPSSs was fabricated by overlapping the MXene film on the PET substrate, after the Ti_3_C_2_T_x_ flakes self-assembling at the liquid interface. Subsequently, the transmittance and absorption of the TPSS at UV, VIS, NIR wavelength bend (300 – 1800 nm) were measured by UV-vis-NIR spectrophotometer (UV3600IPLUS). On the other hand, to test the photothermal conversion of the TPSS, temperature rising of the TPSSs with different layers of the MXene film was measured under illumination of 100 kW cm^-2^. The solar energy was provided by a xenon lamp light source system (Zhongjiao jinyuan Co. Ltd. Beijing, China). The surface temperature was recorded by infrared thermal imaging instrument (Fluke Ti480). Then, in order to investigate the temperature rise of TPSS in low temperature environment, we made an environment box by refrigerating compressor, connected with the closed cavity. In addition, to ensure illumination of the light source, part of the environmental box are made of the transparent acrylic board. Last, TPSS is placed in an environmental box, and the anti-icing/fogging performance of the material is tested by changing the environmental temperature, humidity and other conditions.

**Characterizations.** Morphologies of the Ti_3_C_2_T_x_ flakes was analyzed by using a field emission scanning electron microscopy (SEM, Hitachi, S-4800, Japan). Monolayer MXene film was certificated by transmission electron microscope (TEM, FEI Tecnai Osiris) optical microscope (OM, Olympus) and atomic force microscope (AFM, Bruker dimension icon) The sliding angle and surface tension of droplets were measured by an optical surface analyzer (LSA 100) equipped with a whirler. The photographs and the videos were recorded by a digital camera (Sony α6400, Japan). The temperature distribution of PS platform with assistance of 1 sun and a cooling source was measured by Infrared camera (Fluke Ti480).

1. **Calculation of the photothermal conversion efficiency**

The quantitative evaluation of the photothermal conversion efficiency (PCE, *η*) is important. In this work, *η* is calculated with experimental data by using the widely adopted energy balance model originally developed for photothermal nanomaterials. Under continuous illumination, the steady‑state temperature rise Δ*T*_Max_ of the TPSS satisfies:

$$\eta\text{ = }\frac{hS\Delta T_{\text{Max}}-Q_{\text{loss}}}{IS(1-\text{1}\text{0}^{-A_{\lambda}})}$$

Where *h* is the heat transfer coefficient, *S* is the effective heat exchange area, *I* is light power density, *A*_λ_ is the absorbance of the TPSS at the illumination wavelength (or in the solar spectrum range), Q loss accounts for background heating of water and the container. Among that, the product *hS* is determined experimentally from the cooling curve after switching off the light:

$$hS=\frac{mC}{\tau}$$

where m and C are the mass and specific heat capacity of the heated medium (mainly water in our setup), and τ is the time constant obtained by fitting the cooling curve according to:

$$\theta(t)=\frac{T(t)-T_{env}}{{{Tenv}_{max}}^{-t/\tau}}$$

Therefore, we recorded the transient temperature rising of the MXene‑based TPSS under different simulated solar intensities (60–100 mW cm^-2^), in which the cooling time constant τ is about 60.6 s. The total mass and heat capacity of TPSS sample in the illuminated area (4 cm^2^) is 108.3 mg and 1.41 J g^-1^ K^-1^. Substituting these experimentally determined parameters into the above equations, we obtain a photothermal conversion efficiency of 88.3% for the MXene‑based TPSS under 100 mW cm^-2^ illumination.


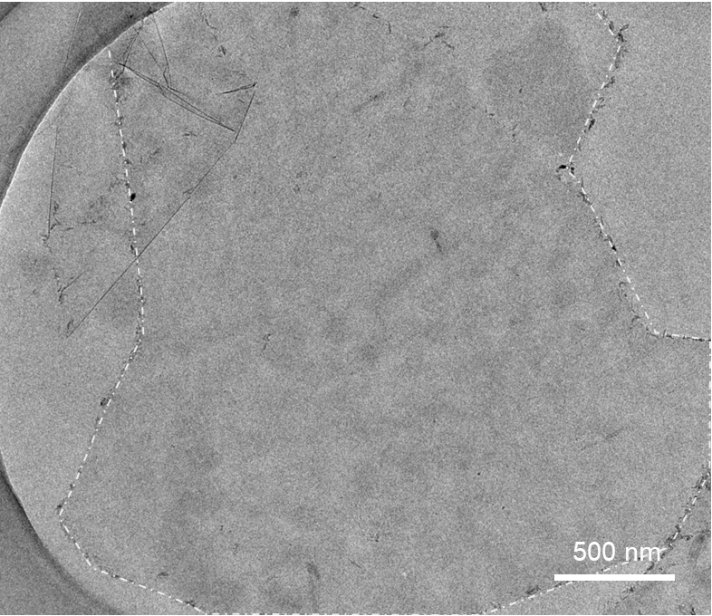


**Figure S1.** TEM of the MXene nanosheets with the single layer.

**
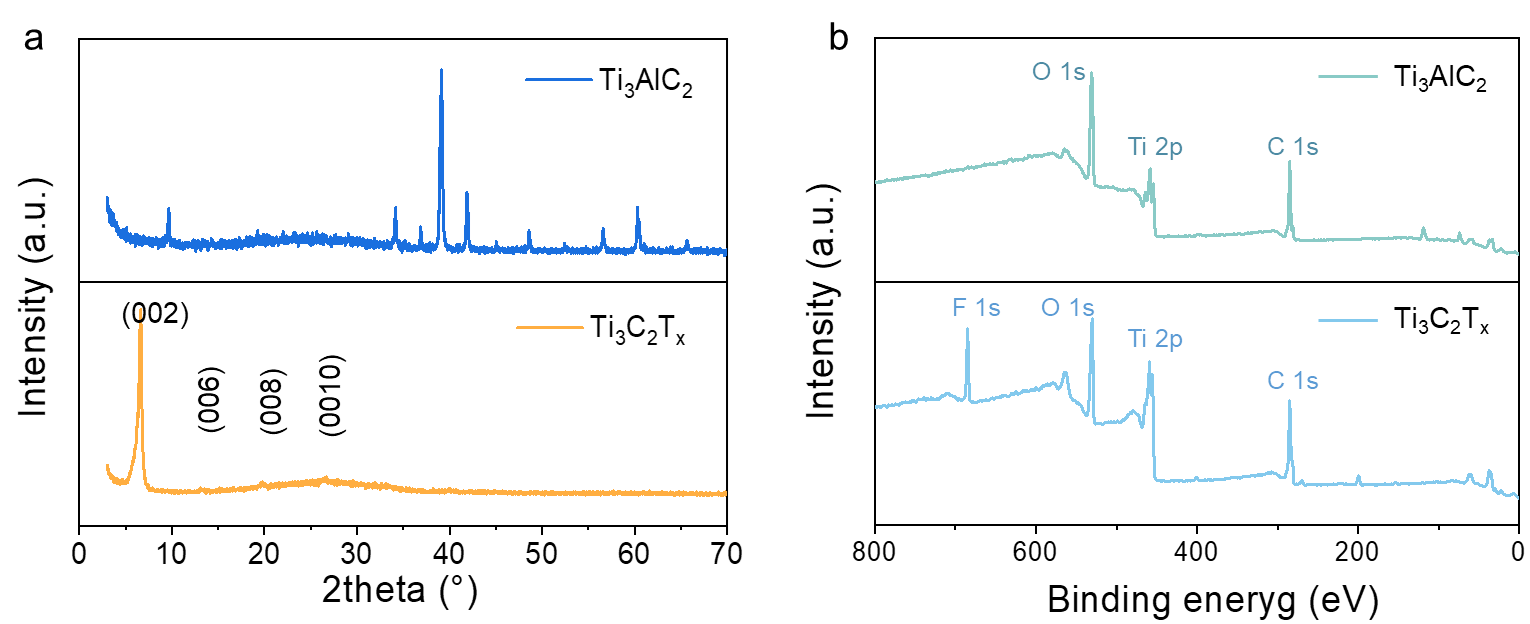
**

**Figure S2.** Characterization of exfoliated Ti_3_C_2_T_x_ nanosheets. (a) XRD and (b) XPS of the Ti_3_AlC_2_ and Ti_3_C_2_T_x_.


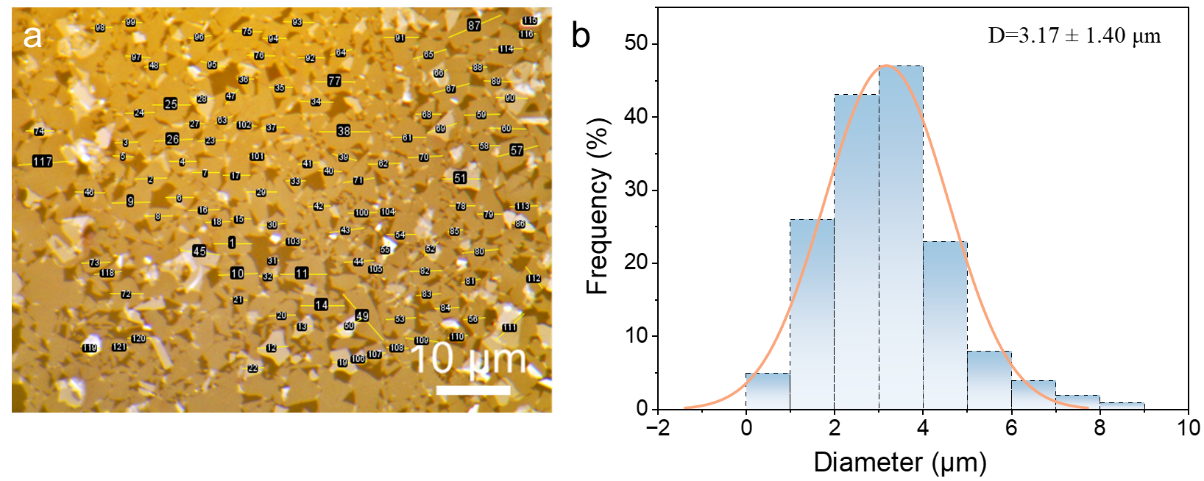


**Figure S3**. Lateral size characterization of Ti₃C₂Tₓ MXene flakes. (a) Optical microscopy image of the self‑assembled MXene film on glass; representative flakes are outlined and labeled to indicate their lateral dimensions. (b) Statistical distribution of the lateral size of MXene flakes obtained from image analysis in (a).


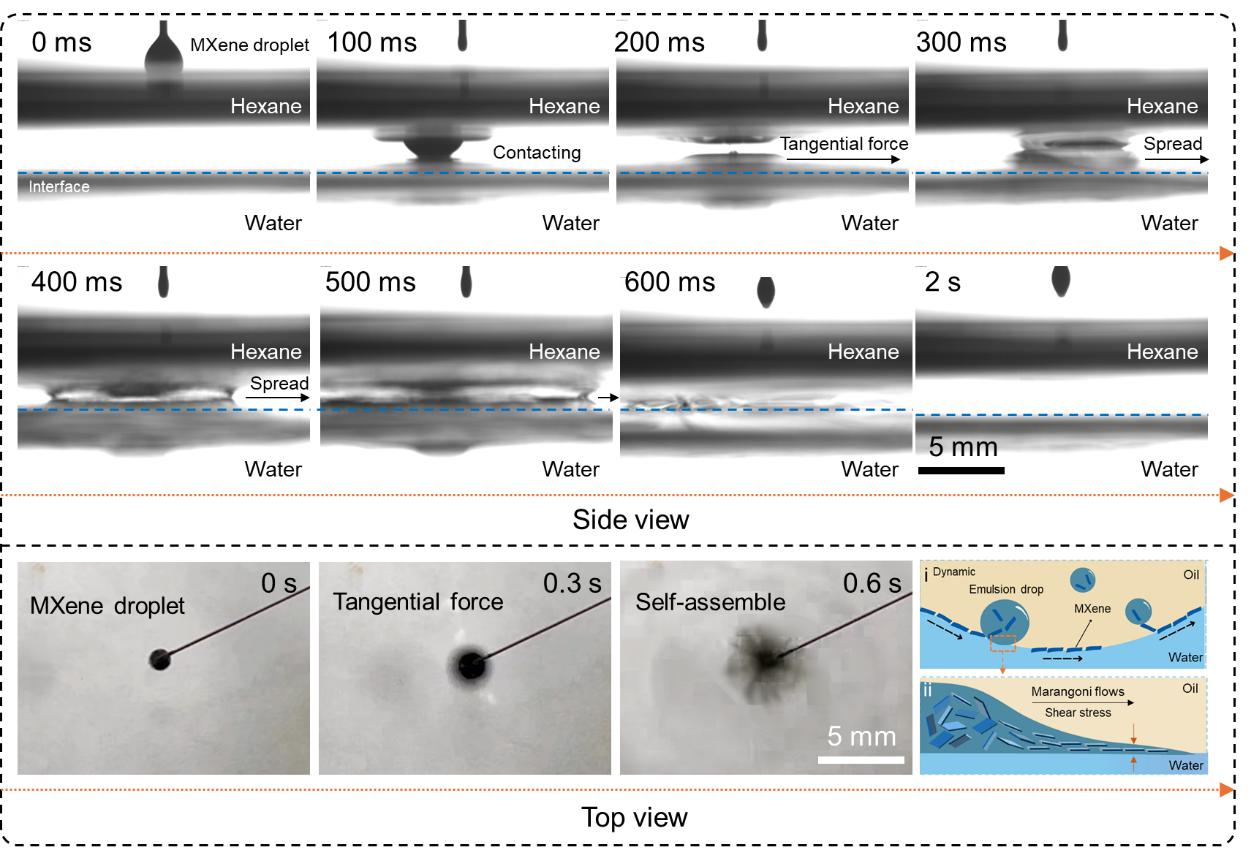


**Figure S4.** Time‑resolved side‑view and top‑view images of an MXene–IPA droplet upon contact with the water/n‑hexane interface. The surface‑tension gradients at the liquid–liquid interface induce Marangoni flow and the associated tangential shear stress, which drives the progressive outward spreading of MXene nanosheets and leads to their directional interfacial self‑assembly into a continuous film.


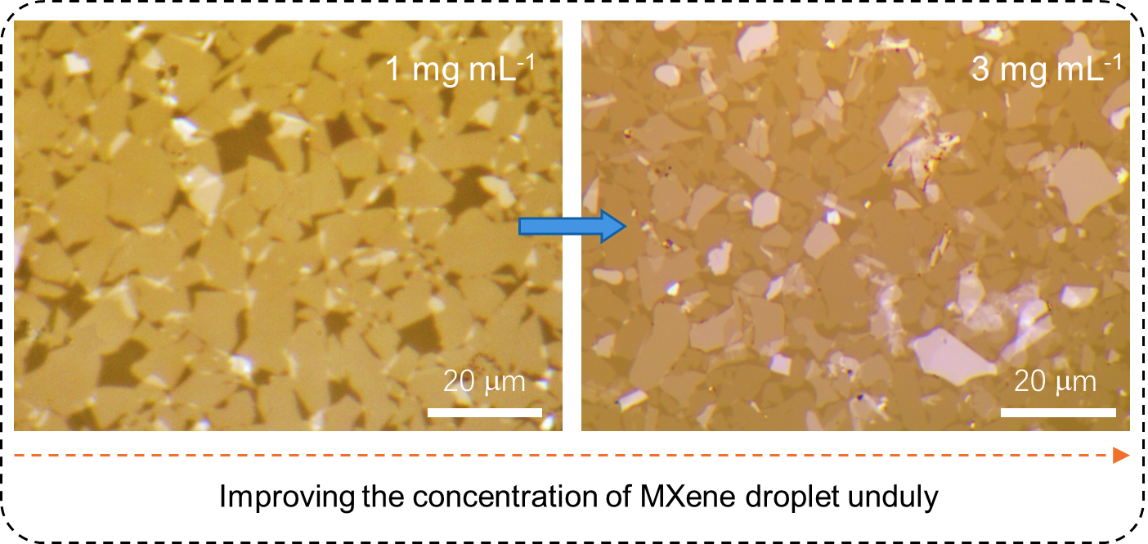


**Figure S5.** Optical microscopy images of MXene films self‑assembled from MXene–IPA solutions with different concentrations. Left: 1 mg mL⁻¹, showing a continuous and uniform monolayer‑dominated film. Right: 3 mg mL⁻¹, where higher MXene concentration leads to increased nanosheet stacking and thickness fluctuations, deteriorating the film uniformity and transparency.


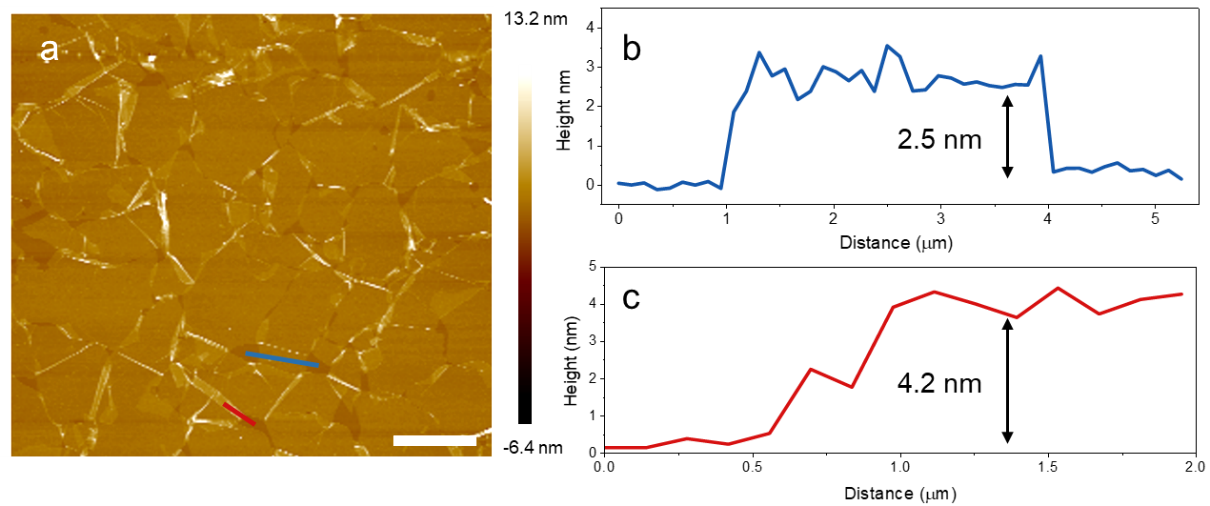


**Figure S6.** (a) AFM image of a MXene self‑assembled film prepared from a 2 mg mL⁻¹ MXene–IPA solution. (b-c) Height profiles along the blue and red lines in (a). The single‑layer regions exhibit a thickness of ≈ 2.5 nm, while the occasional overlapping regions are ≈ 4.2 nm, confirming the good uniformity and homogeneity of the self‑assembled MXene film.


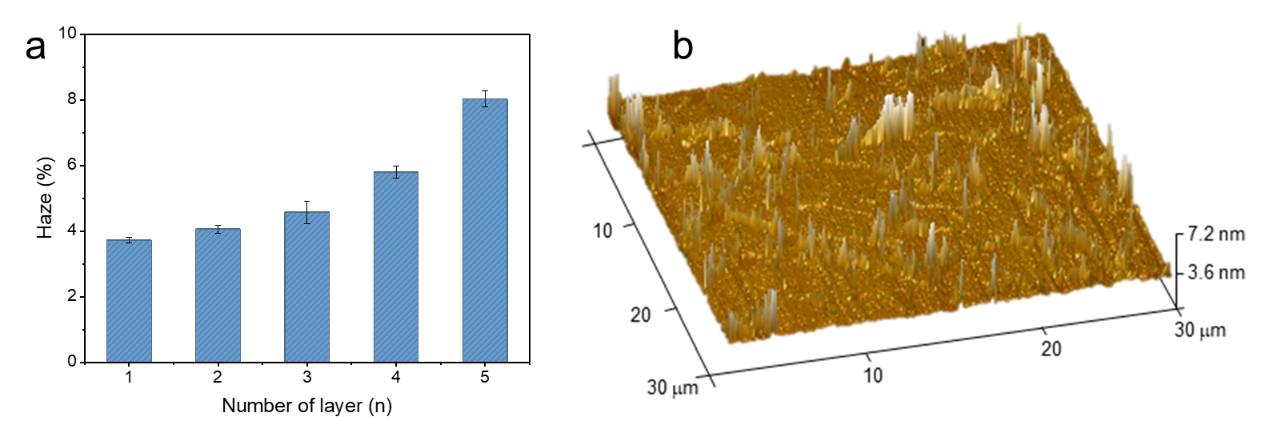


**Figure S7.** (a) Haze of self-assembled MXene films as a function of the number of MXene layers. (b) Three‑dimensional AFM image of the self‑assembled MXene film, showing a smooth and uniform surface with only small thickness fluctuations over a large area.


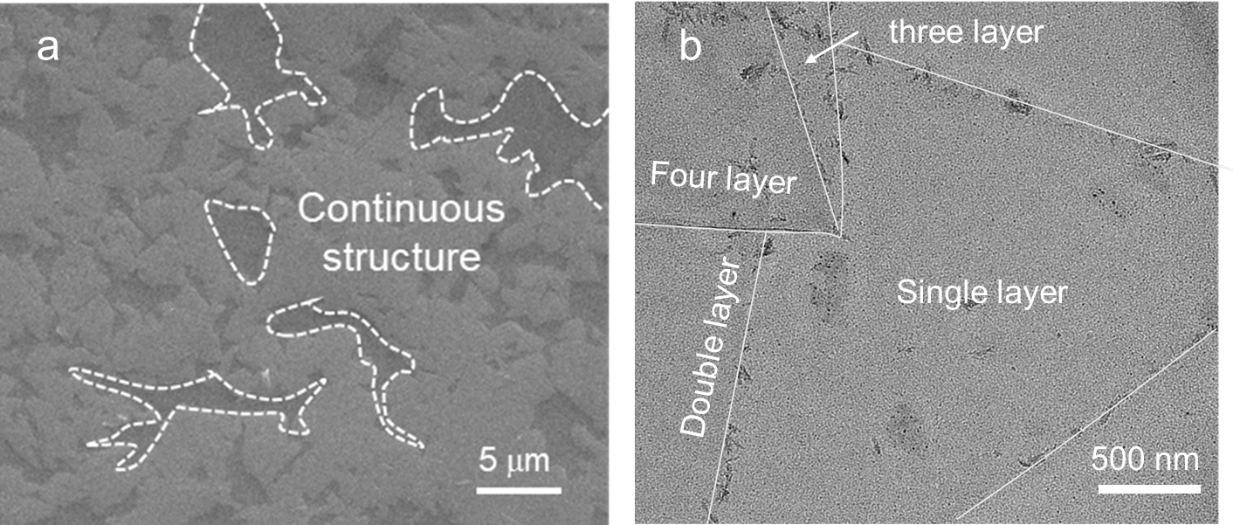


**Figure S8.** (a) SEM image of the ultrathin MXene films by using self-assemble method at liquid interface. It is believed that the inter-bridged MXene network, achieving efficient electrical percolation at a low threshold, facilitates rapid photothermal conversion. (b) HRTEM image of the self-assembled MXene film, revealing the partial stacking structure.


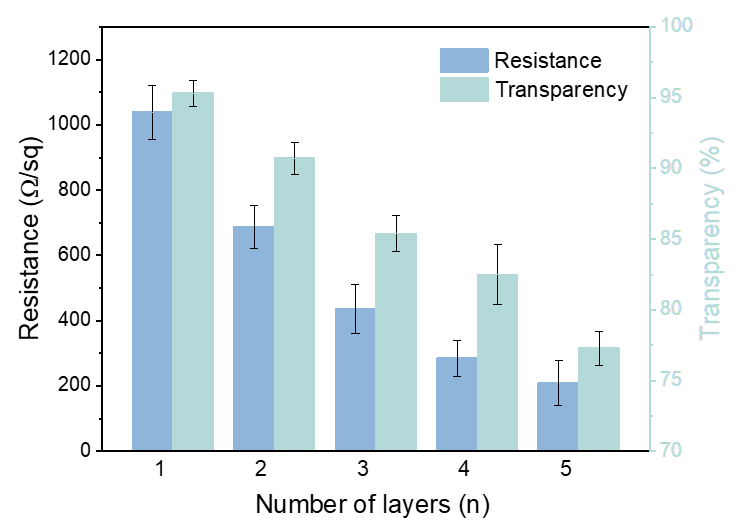


**Figure S9.** Variation of sheet resistance and transmittance of the films with increasing MXene layer number.


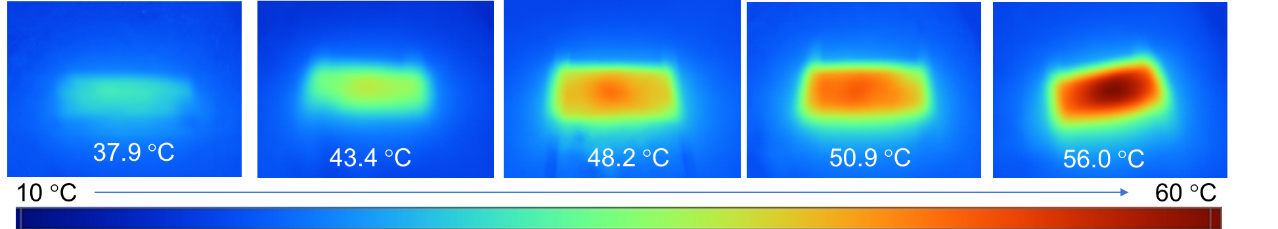


**Figure S10.** Surface temperature of several TPSS with increased MXene layers (1-5 layers) under illumination of 1 sun.


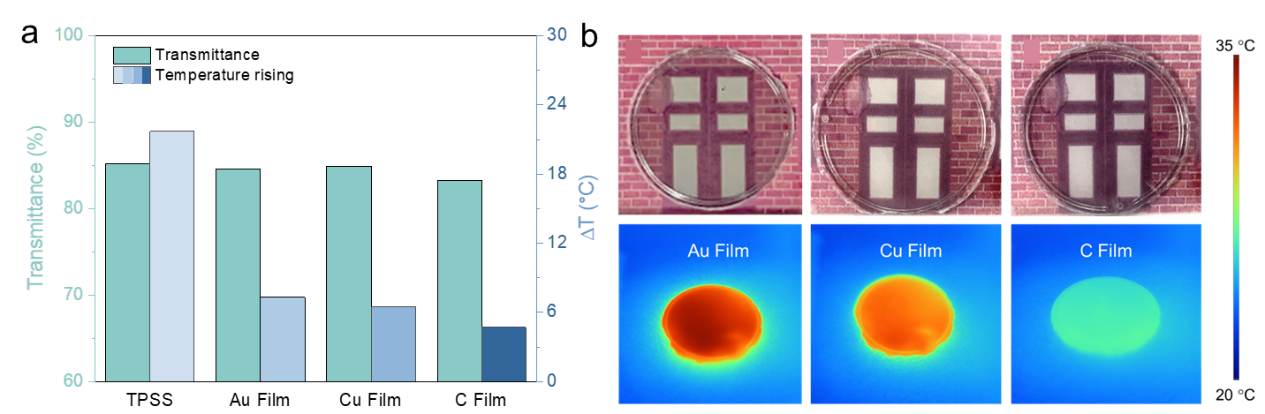


**Figure S11.** (a) The photothermal performance of TPSS and sprayed C, Cu, Au film with the same transparency (80%). (b) the corresponding optical images and surface temperatures of the samples in (a) under illumination of 1 sun.

**
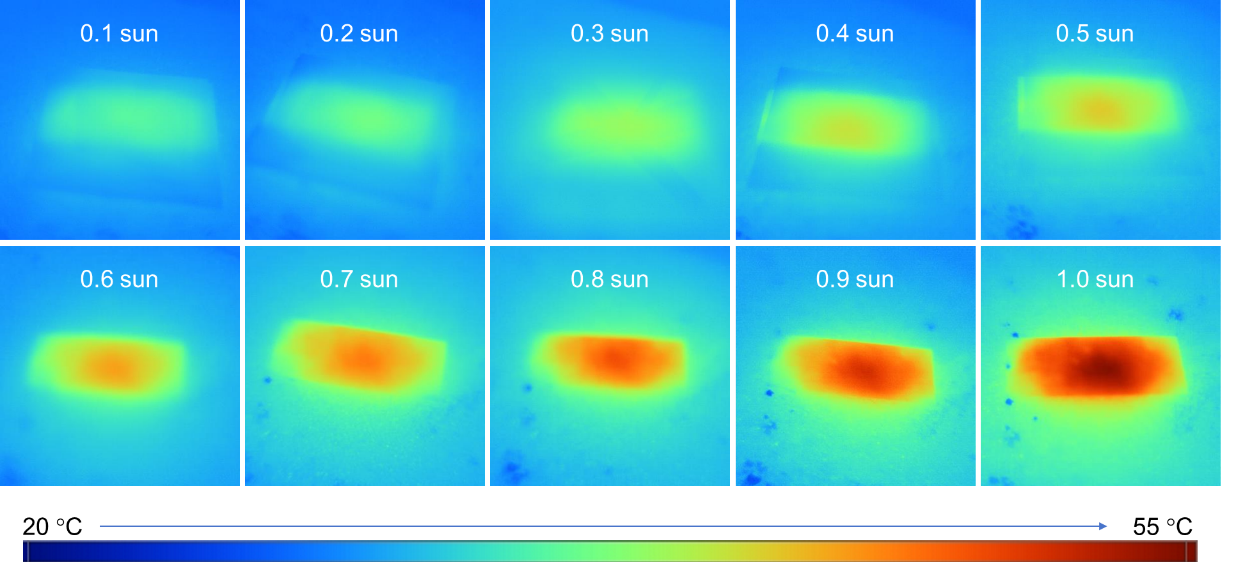
**

**Figure S12.** The surface temperature of TPSS_3_ (with three layers of MXene) under illumination with different light intensity (0.1 - 1 sun).

**
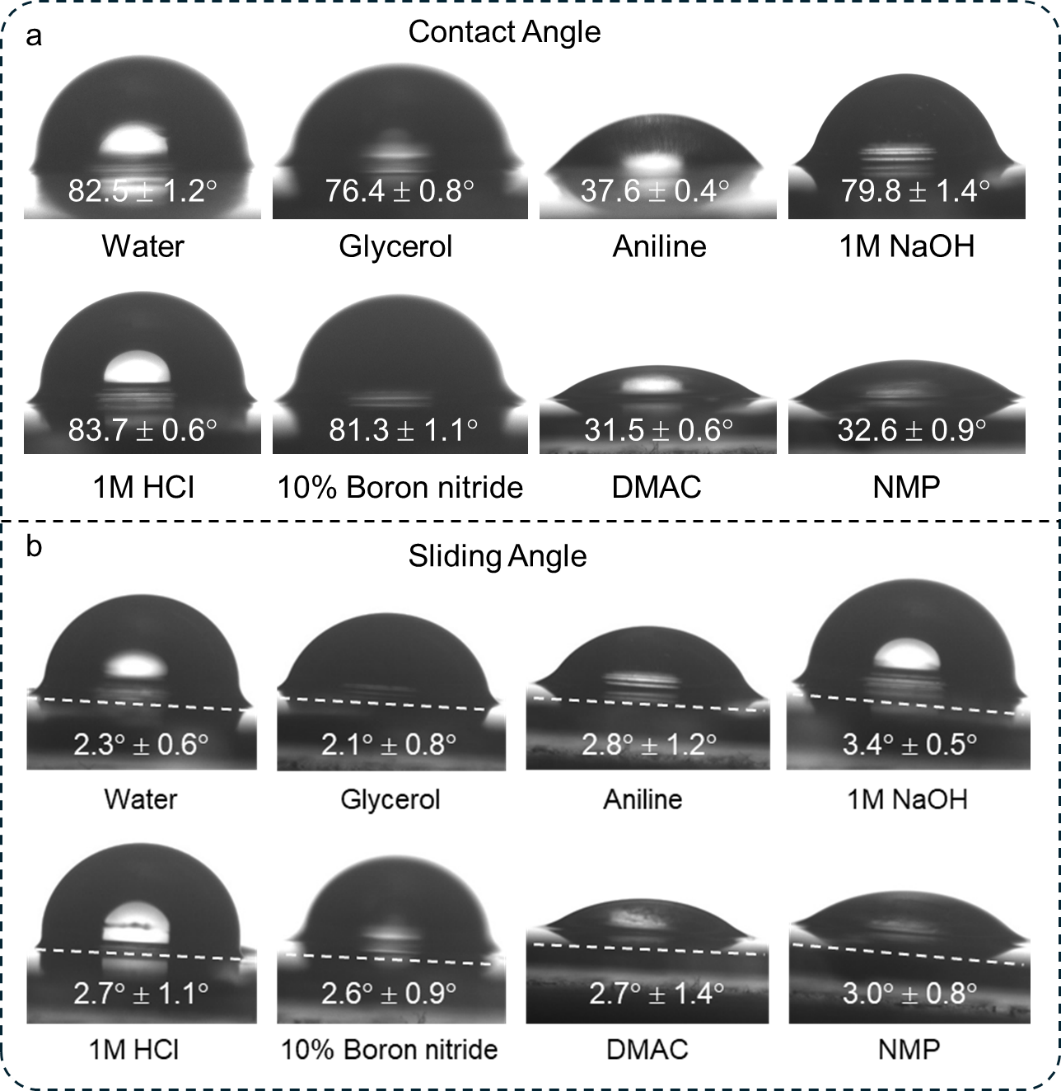
**

**Figure S13.** Sliding angles (SA) of the different liquid on the TPSS, demonstrating the great resistance to various liquids, ranging from water to organic solutions.


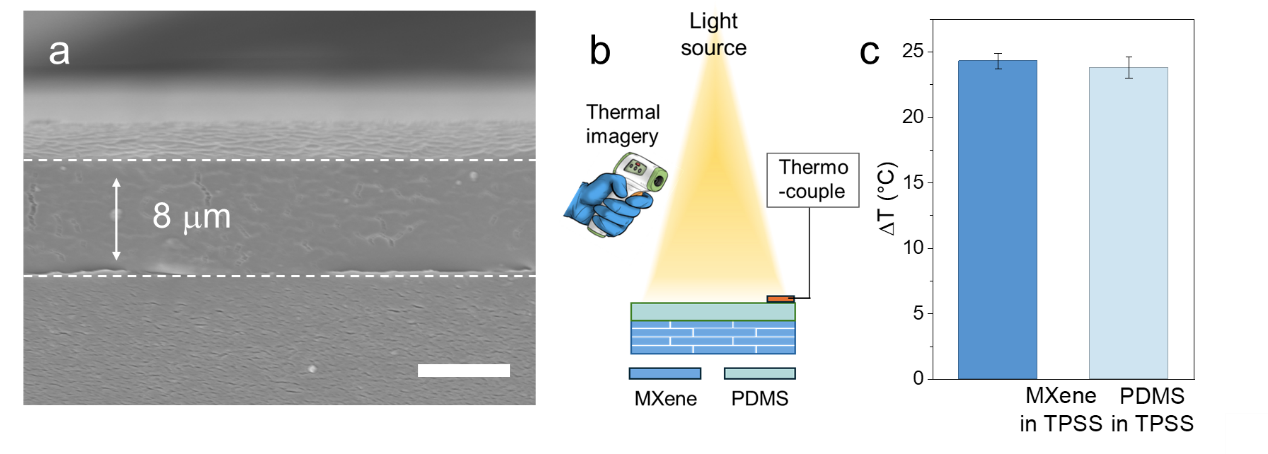


**Figure S14.** Thermal coupling between the MXene photothermal layer and the PDMS slippery surface in TPSS. (a) Cross‑sectional SEM image of the TPSS, showing the ultrathin PDMS slippery overlayer (thickness ≈ 8 μm). (b) Schematic illustration of the measurement setup: the temperature of the internal MXene layer is monitored by infrared (IR) thermography, while the temperature at the outer PDMS surface is measured by a thermocouple attached to the top surface. (c) Comparison of the steady‑state temperature rise (ΔT) of the MXene layer (IR) and the PDMS surface (thermocouple) under 100 mW cm⁻² illumination, showing that the temperature difference across the thin PDMS layer is less than 1 °C.


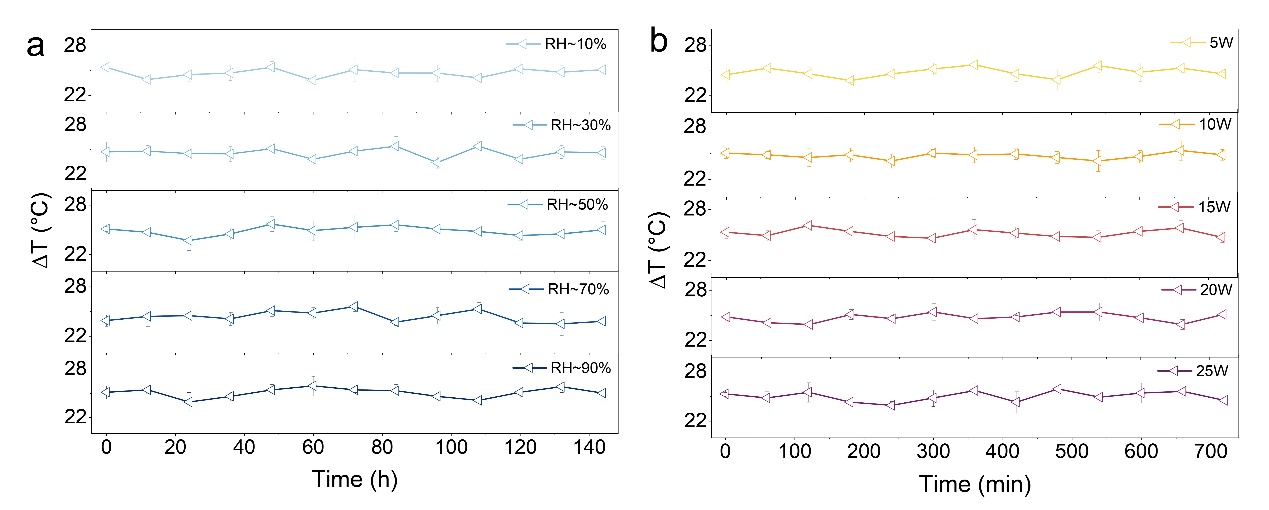


**Figure S15.** Environmental stability of TPSS under humidity and UV irradiation. (a) Photothermal temperature rise (ΔT) of TPSS under 1‑sun illumination after storage at different relative humidities (10–90% RH, 25 °C) for up to 144 h, demonstrating stable photothermal performance and no obvious degradation. (b) ΔT of TPSS during continuous UV irradiation (365 nm) at different lamp powers (5–25 W) for up to 720 min, showing excellent UV stability with only minor fluctuations in temperature rise and no visible damage to the surface.


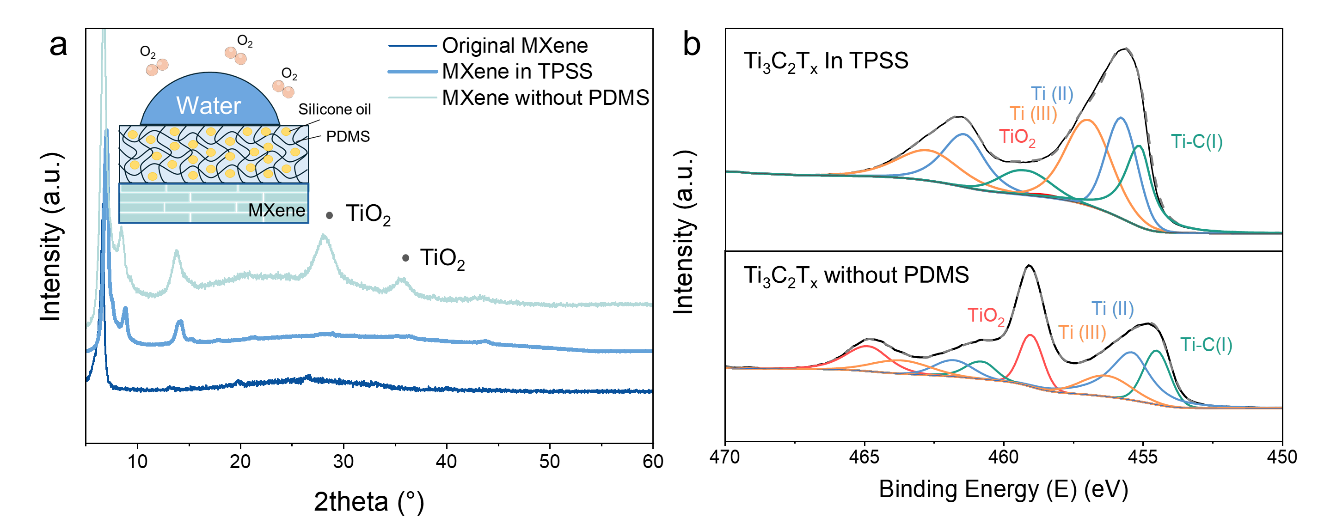


**Figure S16.** Structural and chemical stability of MXene with and without TPSS protection under humid conditions.(a) XRD patterns of the original Ti₃C₂Tₓ film, Ti₃C₂Tₓ embedded in TPSS, and Ti₃C₂Tₓ without PDMS after exposure to 90% RH for 144 h. The inset schematically illustrates the protection of the MXene layer by the slippery surface. (b) Ti 2p XPS spectra of Ti₃C₂Tₓ in TPSS (top) and Ti₃C₂Tₓ without PDMS (bottom) after the same treatment.


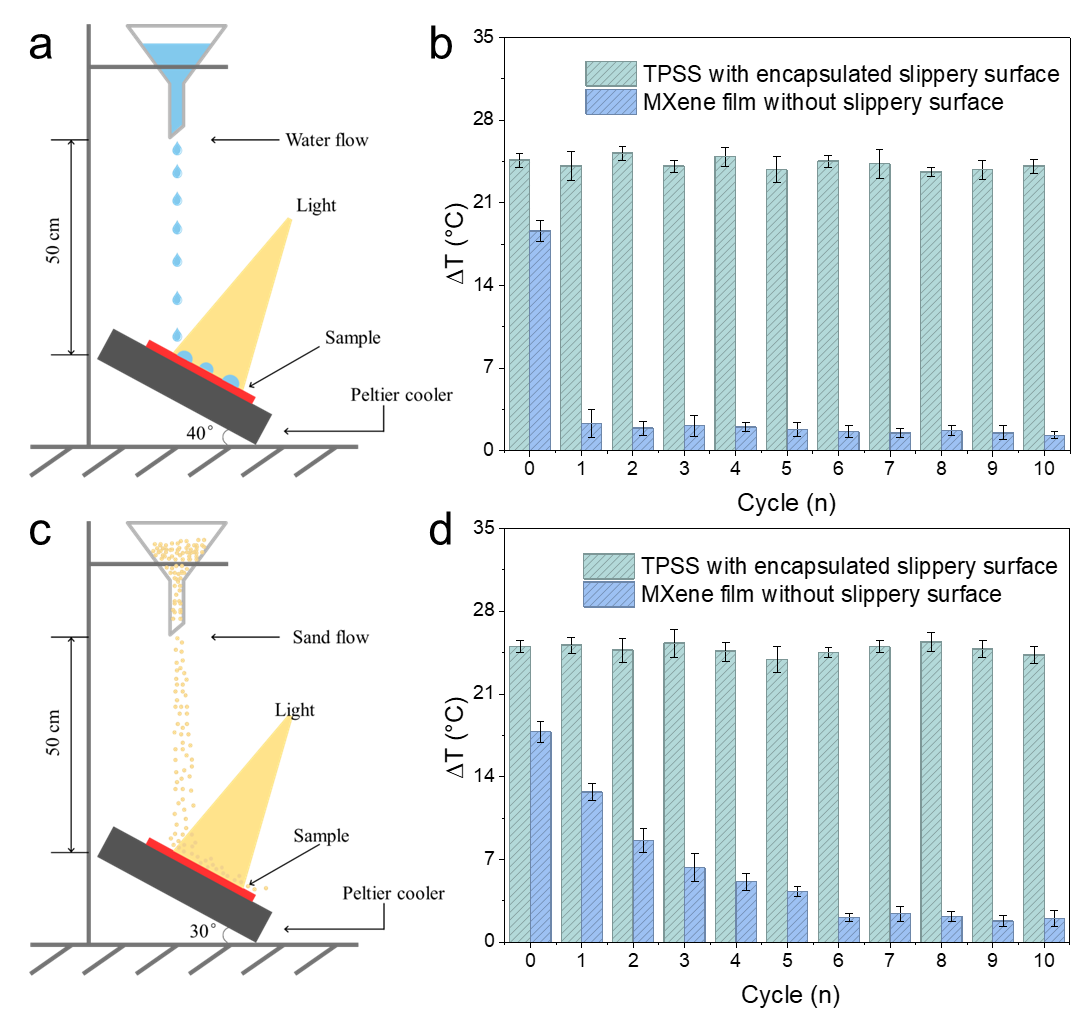


**Figure S17.** Durability of TPSS under simulated rain and sand impact. (a) Schematic illustration of the rain‑droplet impact test, in which deionized water is dripped onto the inclined sample to mimic heavy rain conditions. (b) Photothermal temperature rise (ΔT) of TPSS and bare MXene films (without PDMS slippery protection) as a function of rain‑impact cycles under 1‑sun illumination. TPSS maintains an almost constant ΔT, whereas the hydrophilic ultrathin MXene film is progressively damaged and loses its photothermal performance. (c) Schematic illustration of the sand/dust impact test, where quartz sand particles are continuously dropped onto the inclined sample to simulate sandstorm erosion. (d) ΔT of TPSS and bare MXene films versus sand‑impact cycles. TPSS shows nearly unchanged photothermal performance, while the unprotected ultrathin MXene film is severely abraded, leading to rapid failure of the photothermal function.


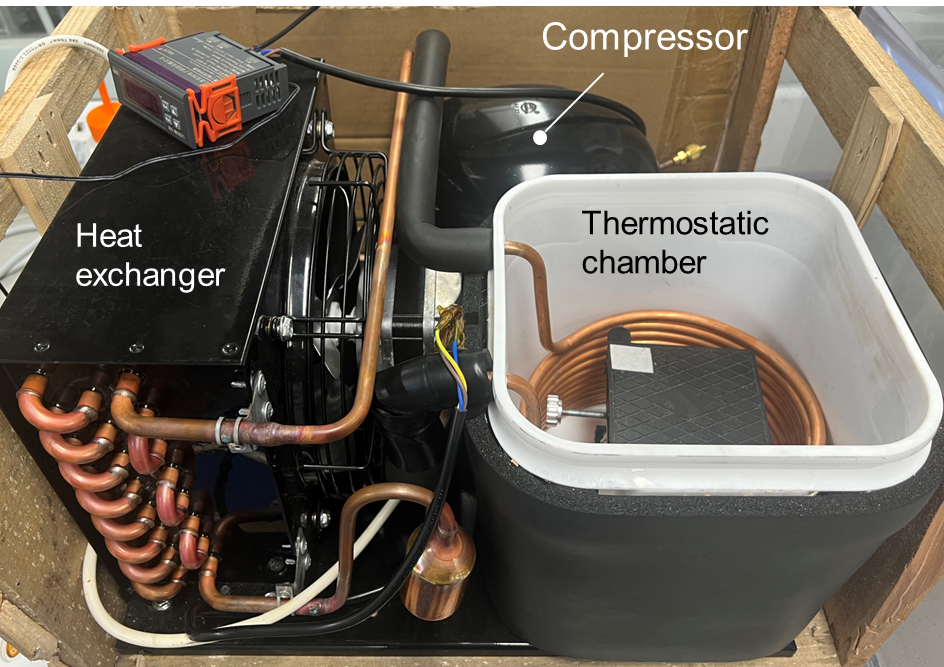


**Figure S18.** Photo of the home-made thermostatic chamber with low temperature to evaluate the anti-icing/fogging performance of TPSS.


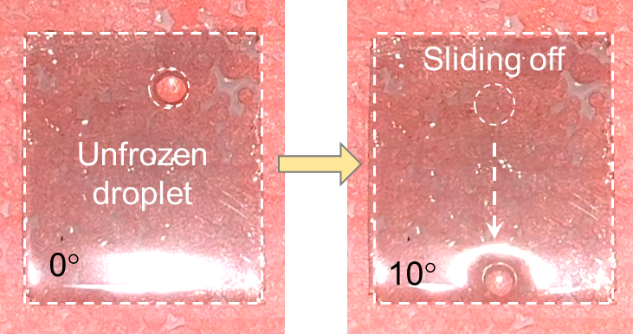


**Figure S19.** Photo of the unfrozen droplet at -20 °C sliding off the TPSS with the inclined angle of 10°.


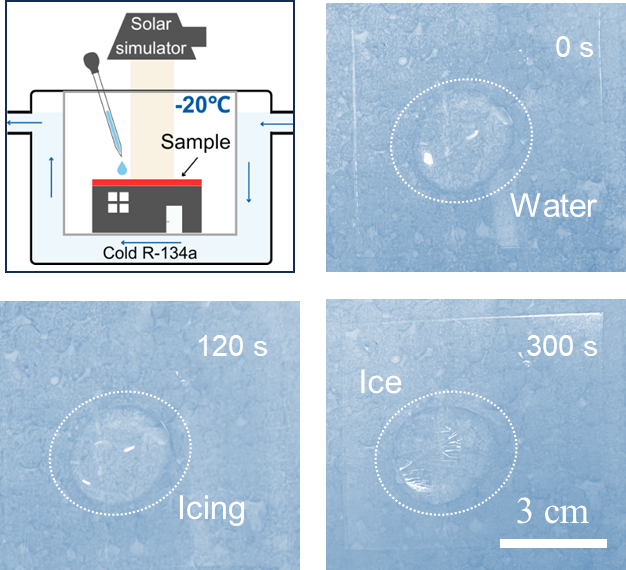


**Figure S20.** A sequential photographic series showing the icing process of supercooled water droplets upon SLIPS without transparent MXene film.


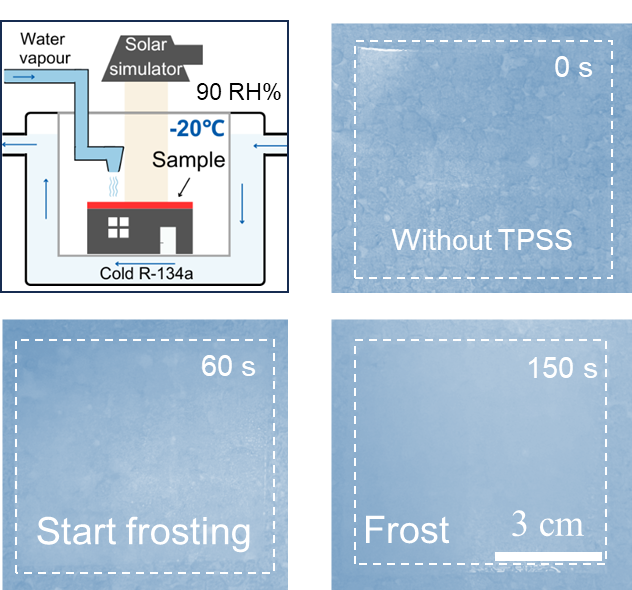


**Figure S21.** A sequential photographic series showing the frosting process of the SLIPS without transparent MXene film under high humidity (90%).


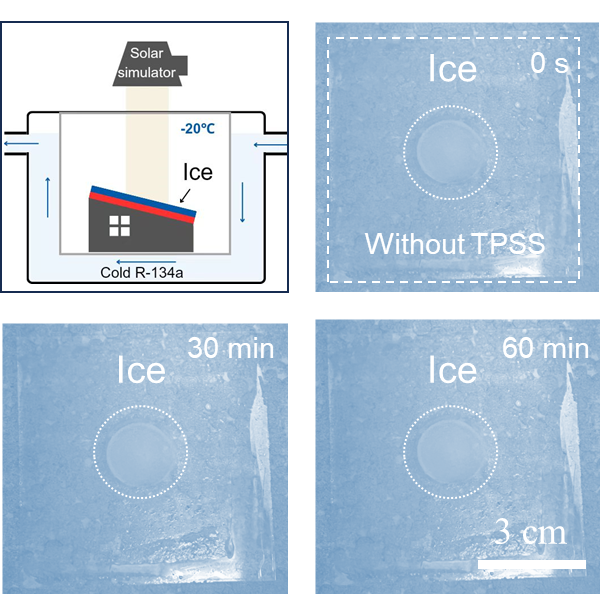


**Figure S22.** A sequential photographic series showing the failure deicing performance of the slippery surface without transparent MXene film at -20 °C.


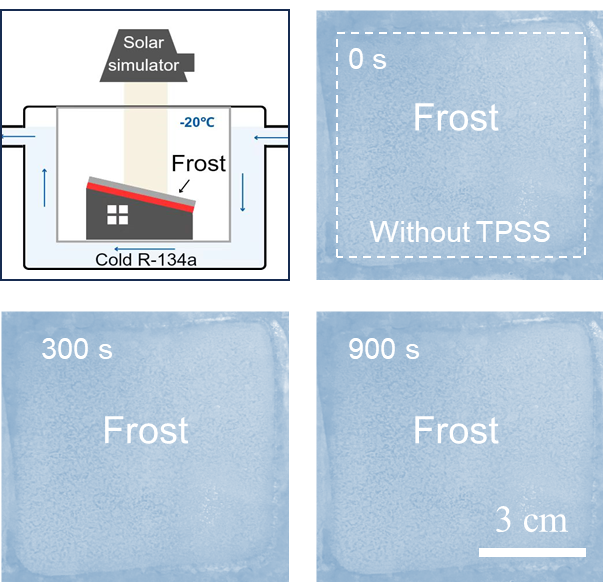


**Figure S23.** A sequential photographic series showing the failure de-frosting performance of slippery surface without transparent MXene film at -20 °C.
